# The effect of 12-week high-dose *Colostrum Bovinum* supplementation on immunological, hematological and biochemical markers in endurance athletes: a randomized crossover placebo-controlled study

**DOI:** 10.3389/fimmu.2024.1425785

**Published:** 2024-10-21

**Authors:** Krzysztof Durkalec-Michalski, Natalia Główka, Tomasz Podgórski, Małgorzata Woźniewicz, Paulina M. Nowaczyk

**Affiliations:** ^1^ Department of Sports Dietetics, Poznan University of Physical Education, Poznań, Poland; ^2^ Sport Sciences–Biomedical Department, Charles University, Prague, Czechia; ^3^ Department of Physiology and Biochemistry, Poznan University of Physical Education, Poznań, Poland; ^4^ Department of Human Nutrition and Dietetics, Poznań University of Life Sciences, Poznań, Poland

**Keywords:** immunity, immunonutrition, sports nutrition, triathlon, ergogenic support, proteins

## Abstract

**Background:**

*Bovine colostrum* (COL) is assumed to be one of the strongest natural immune stimulants. Regular ingestion of COL may contribute to improved immune response in athletes exposed to high training loads.

**Methods:**

Twenty-eight endurance-trained males aged 31.1 ± 10.2 years (body mass 81.9 ± 9.0 kg; height 1.82 ± 0.06 m) completed this randomized double-blind placebo(PLA)-controlled crossover study aimed at investigating the effect of 12-week COL supplementation (25g_COL_·day^-1^) on resting (REST), exercise-induced (POST-EX), and short-term post-exercise recovery (REC; 1 h after test exercise) changes in selected saliva and blood immunoglobulins (Ig), white blood cell (WBC) count and differential; as well as blood hematological, nutritional status and muscle damage indices. The protocol assumed 4 study visits – before/after supplementation with COL (*COL_PRE_* and *COL_POST_*) and PLA (*PLA_PRE_* and *PLA_POST_*). During testing sessions, incremental rowing test to exhaustion and swimming-specific performance test were introduced as exercise stimuli.

**Results:**

At *COL_POST_* visit the secretory IgA (SIgA) concentration in saliva was significantly higher at POST-EX and REC compared to REST (*p*<0.05). COL supplementation had no effect on blood IgA, IgE, IgD, IgG, and IgM concentrations. Furthermore, after COL supplementation decrease of hematocrit at REC (*p*<0.05) was revealed.

**Conclusions:**

12-week supplementation with 25 g_COL_·day^-1^ in endurance-trained male athletes resulted in a favorable increase in post-exercise concentration of salivary SIgA. COL seems to be a potential stimulator of local immune defense after exercise-induced homeostasis disturbances. Nevertheless, the lack of effect on blood markers indicates the need for further research in the area of mechanisms underlying the effect of the supposed COL immunological capacity.

## 1 Introduction

Moderate and recreational physical activity may improve the functioning of the immune system and reduce the risk of infections. Nevertheless, evidence-based research studies confirm that certain physically stressed groups, e.g. endurance athletes (mainly swimmers and triathletes) involved in prolonged and/or intensive physical training may be more susceptible to bacterial and viral infections. It has been observed that some components of the immune system are suppressed after exercise, which can last from a few hours to even a few days. Exercise*-*induced immune disturbances may contribute to compromised well-being, health, physical capacity, and training/competition performance (1–4). Thereby, it is essential to explore different strategies to improve the immunological capacity, like nutrition or supplementation.

The impact of exercise concerns different types of immunity (1). Perturbations are especially seen in the number of circulating leukocytes, and it has been confirmed that leukocytosis may occur during and post-exercise (5, 6). An increase in the neutrophils:lymphocyte ratio may serve as an indicator of the overall stress response immediately after exercise (1). Moreover, at the recovery phase, lymphocytopenia can also be observed (1). Sports studies have also revealed a possible decrease in immunoglobulin (Ig) G_2_ associated with exercise (7). It has been shown that a decreased IgG_2_ concentration may be associated with an increased bacterial infection risk. There is also a strong positive correlation between IgG_2_ and the ability to produce antibodies (8). Furthermore, the common mucosal immune system is considered to be the first line of defense, while local production of secretory IgA (SIgA) in saliva is recognized as the major effector of this system (1). Athletes who suffer from SIgA deficiency may contract upper respiratory tract infections (URTI) regularly (9). In turn, an increase in SIgA may be the primary mechanism for the decreased URTI risk (10).

Among the various supplements, *Colostrum Bovinum* (COL) seems to induce beneficial effects *via* the improvement of immune function. COL is a substance produced naturally by the cows’ mammary glands for 24–72 h after calving. The significant impact of COL ingestion on the development of the calves’ immune system has led to the use of COL-based products in humans (11–14). Evidence suggests that COL may have many clinical or therapeutic applications in humans (15). It contains more biologically active compounds, higher concentration of lactoferrin, and 100-fold higher concentrations of Igs than in mature milk (14, 15). For the adult human, COL supplementation is considered to be well tolerated and safe, with only mild, possible adverse effects, like nausea, diarrhea, flatulence, unpleasant taste, abdominal discomfort, which may disappear with time. Unfortunately, there is no existing data for long-term use of COL, therefore no conclusions on the effect of COL supplementation on immune function can be currently made (15, 16). In the previously published meta-analysis (16), it was shown that COL may have certain positive effects in reducing the rate of URTI days and episodes. In turn, our recently published meta-analysis (17), focused on the most commonly reported immunological markers in COL supplementation studies on physically active people to consider their significance in explaining previously reported effects regarding URTI incidence. Nevertheless, diversity in the supplementation strategies (supplementation dosages [10–20 g_COL_·day^-1^]; supplementation duration, or spreading the dose), as well as sample size, time of blood and saliva collection, and lack of evaluations of numerous clinically specific immunological markers, renders the comparison between interventions difficult. Eventually, no clear conclusions on the effect of COL supplementation on immunological outcomes can be made.

Moreover, several strategies have been investigated to mitigate exercise-induced muscle damage, which may potentially hinder training adaptations. Supplementation with protein- and amino acid-based products has been considered one of the strategies in addressing these specific areas of concern (18). It is confirmed that adding protein to the diet may suppress the rise in plasma proteins linked to myofibrillar damage, and thus can help to maintain a favorable anabolic hormone profile or minimize increases in muscle damage (19). COL, as the “first” milk, is rich in proteins (14) and therefore may be considered a protein source valuable especially in terms of muscle adaptations and nutritional status.

Although different kinds of protein supplements in sports are well-studied, and widely used in athletes, data on high-dose COL effect in exercise conditions on biochemical indices are scarce. Therefore, the aim of the current study was to evaluate the effect of chronic 12-week high-dose COL supplementation (25 g_COL_·day^-1^) on the saliva and blood biochemical indices in regards to different time points: at rest, post-exercise and short-term recovery in a group of healthy, moderately endurance-trained males participating in triathlon and swimming training. We investigated the effect of COL on the concentrations of Igs in saliva and blood, as well as white blood cell (WBC) count and differential (primary outcomes). We additionally investigated hematological, muscle damage, and nutritional status indices in blood (secondary outcomes). We hypothesized that COL supplementation will prevent exercise-induced unfavorable disturbances in SIgA in saliva and IgG in blood. Additionally, we hypothesized that COL will induce lower leukocytosis immediately post-exercise and lower lymphocytopenia in the recovery state. Moreover, it was assumed that hematological, nutritional status indices and muscle damage markers will not be affected after COL supplementation in comparison to milk protein used as the placebo (PLA) control.

## 2 Materials and methods

### 2.1 Study participants

**Figure 1:**
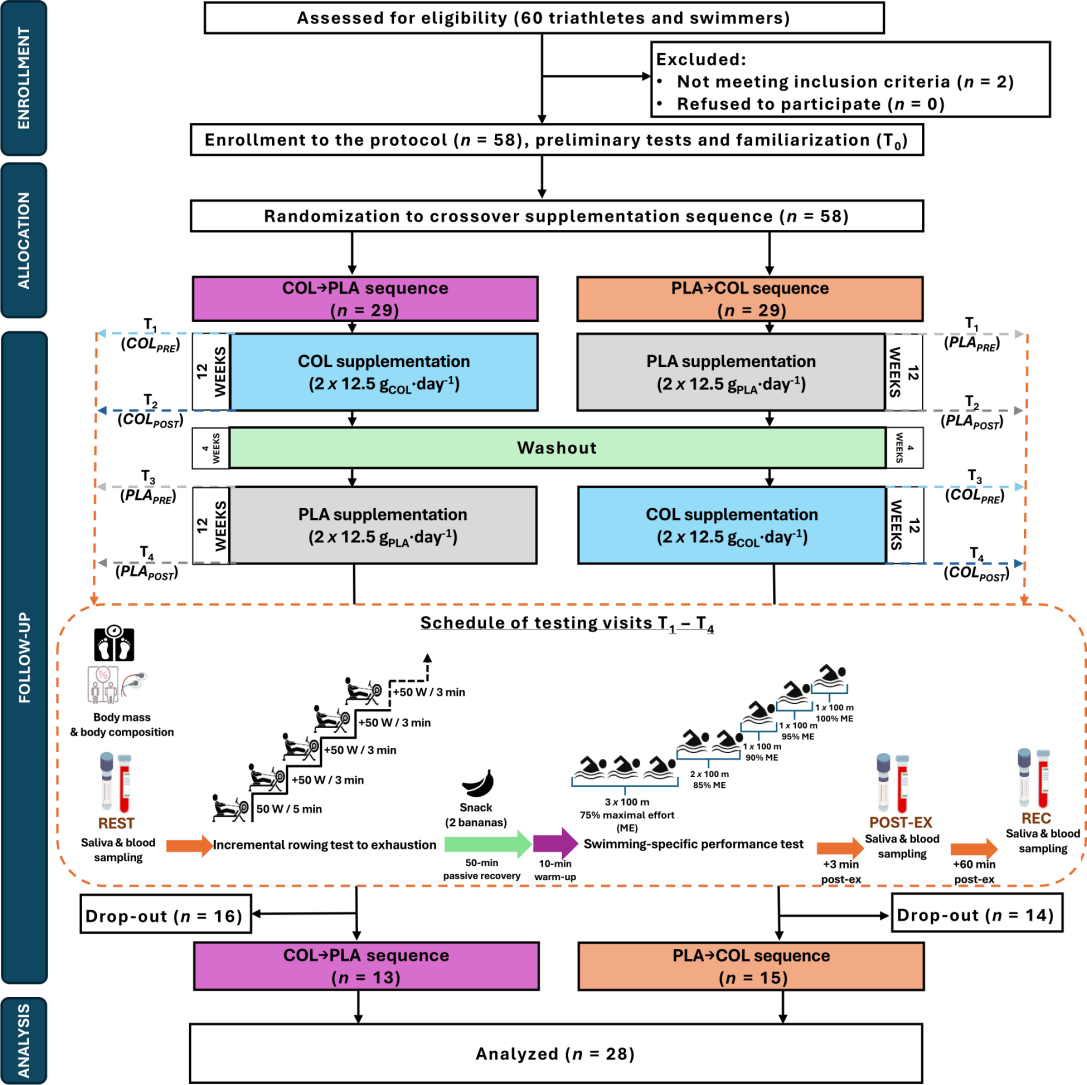
Flowchart of the study design.

Fifty-eight moderately endurance-trained male participants were initially enrolled in this study. There were 30 dropouts from the study protocol (**Figure 1**). The main reasons for dropping out were: injuries (*n*=8), antibiotic therapy during the protocol (*n*=4), business trips (*n*=3), family reasons (*n*=3); as well as termination without providing the reason during the washout period (*n*=12). There were 5 dropouts during COL and 6 dropouts during PLA supplementation periods. Finally, 28 athletes (31.1 ± 10.2 years; 81.9 ± 9.0 kg body mass; 1.82 ± 0.06 m height) completed the entire study protocol and were included in the analyses (**Figure 1** and **Table 1**). All athletes were members of the sports clubs from Poland (mainly Poznań, Szczecin, Wrocław), from which 17 were triathletes and 11 were swimmers. The inclusion and exclusion criteria of the enrolled participants were checked by a medical professional. The criteria for qualifying for the study included good health condition without chronic health disorders, a valid and up-to-date medical certificate confirming the athlete’s ability to practice sports, at least 5 years of triathlon/swimming training experience, at least 3–5 training units per week (and the declaration of performing that number of workouts during both supplementation periods and washout period), regular participation (at least 2–3 times per year) in triathlon/swimming competitions on at least national level. The exclusion criteria were allergy to cow’s milk proteins, lactose intolerance or any co-existing autoimmune diseases, reporting symptoms of infection, or taking any medication for 4 weeks before enrollment to the study protocol. The study protocol was conducted in a few waves from November 2021 to May 2023 at the Department of Sports Dietetics (Poznan University of Physical Education, Poland). Each of the waves started during autumn/winter months and was completed during spring/summer months. Thus, the number of participants randomized to ingest COL and PLA during autumn/winter months (higher risk of URTI) was equal (of analyzed athletes, 13 were randomized to COL→PLA and 15 to PLA→COL supplementation sequence). All athletes declared that they had not introduced any changes in their lifestyles, usual elements of training, nutrition, or supplementation during the study protocol and that they were prepared for each study visit in the same manner. Habitual diet of participants was evaluated before each of main study visits using 3-day dietary recording. Participants were trained in dietary recording during familiarization visit. Dietary records were discussed during each main study visits with each study participant. The results are presented in the **Supplementary Table 1**. The results indicated proper compliance with dietary recommendations, and stable energy value and macronutrient intake across measuring time points in the study participants. Among study participants 12 were supplementing creatine, 11 whey protein, and 6 beta-alanine. Those participants declared to use the supplements at the constant dose during the entire study protocol – during both supplementation periods, as well as during washout period. Moreover, all participants reported regular use of isotonic drinks during participation in the study protocol. This trial was reviewed and approved by the Bioethics Committee at Poznan University of Medical Sciences (reference number 486/19, issued on April 11, 2019) and was registered retrospectively at ClinicalTrials.gov (NCT06390670). The study complies with the CONSORT Statement for randomized trials as shown in **Figure 1** and **Supplementary Table 2**. All study participants gave written informed consent. All procedures were carried out in accordance with the ethical standards of the Helsinki Declaration of 2013.

**Table 1:** Baseline characteristics of study participants.

Indicator	Units	All	COL→PLA sequence	PLA→COL sequence	*p* COL→PLA *vs.* PLA→COL
		*n* = 28	*n* = 13	*n* = 15	
**Age**	**(years)**	31.1 ± 10.2 (27.2 – 35.1)	31.2 ± 11.3 (24.4 – 38.1)	31.1 ± 9.5 (25.8 – 36.4)	0.967
**Body mass**	**(kg)**	81.9 ± 9.0 (78.4 – 85.4)	80.8 ± 6.1 (77.1 – 84.5)	82.8 ± 11.0 (76.7 – 88.9)	0.560
**Height**	**(m)**	1.82 ± 0.06 (1.80 – 1.84)	1.82 ± 0.06 (1.78 – 1.86)	1.82 ± 0.07 (1.78 – 1.86)	0.907
**Total body water**	**(%)**	58.5 ± 5.2 (56.4 – 60.5)	57.8 ± 4.4 (55.1 – 60.5)	59.0 ± 5.9 (55.8 – 62.3)	0.536
	**(L)**	47.7 ± 4.2 (46.1 – 49.3)	46.6 ± 3.8 (44.3 – 48.9)	48.7 ± 4.4 (46.2 – 51.1)	0.196
**Fat-free mass**	**(%)**	84.1 ± 5.5 (81.9 – 86.2)	84.2 ± 4.3 (81.7 – 86.8)	83.9 ± 6.5 (80.3 – 87.5)	0.869
	**(kg)**	68.5 ± 6.2 (66.2 – 70.9)	67.9 ± 5.2 (64.8 – 71.0)	69.0 ± 7.1 (65.1 – 73.0)	0.644
**Fat mass**	**(%)**	16.0 ± 5.4 (13.9 – 18.1)	15.8 ± 4.1 (13.3 – 18.3)	16.1 ± 6.5 (12.5 – 19.7)	0.889
	**(kg)**	13.3 ± 5.5 (11.2 – 15.5)	12.8 ± 3.8 (10.6 – 15.1)	13.8 ± 6.7 (10.1 – 17.5)	0.658
**Time of the last 100-meter-long section of SSP test**	**(s)**	85.64 ± 21.07 (77.47 – 93.81)	83.25 ± 21.73 (70.12 – 96.39)	87.70 ± 21.01 (76.07 – 99.34)	0.587
**Maximal oxygen uptake**	**(mL·min^-1^·kg^-1^)**	51.1 ± 7.5 (48.0 – 54.1)	51.2 ± 7.4 (46.4 – 55.9)	51.0 ± 7.9 (46.4 – 55.6)	0.963

The results are expressed as the mean ± standard deviation and 95% confidence interval (in parentheses). SSP, swimming-specific performance. The data were analyzed with *T*-test for independent variables (according to the sequence of treatment subgroups: COL→PLA vs. PLA→COL).

G*Power software (version 3.1.9.4, Universität Düsseldorf, Germany) was used to calculate the sample size required to obtain a power of approximately 80% (α = 0.05) and large effect size partial eta squared 0.14 in the analysis of variance (ANOVA) with repeated measurements (RM) within-between factors. Analysis indicated that a sample size of 26 would be suitable for detecting a difference between four measurements.

### 2.2 Study design and visits

The study protocol consisted of a 12-week COL or PLA supplementation in a randomized double-blind crossover design. Crossover design was implemented to avoid potential bias derived from relatively high inter-individual physiological diversity in resting concentrations and diurnal patterns of excretion of the evaluated herein saliva and blood immunological outcomes. Comparison between the same group of participants in two supplementation periods (crossover), seem to generate lower variability in resting immunological outcomes than comparison of two distinct groups of participants (parallel design). The entire study protocol included familiarization and four main visits to the laboratory (T_1_–T_4;_ before/after supplementation with COL [*COL_PRE_* and *COL_POST_*] and PLA [*PLA_PRE_* and *PLA_POST_*]) (**Figure 1**). T_1_ and T_3_ were pre-supplementation (baseline) visits. After the familiarization to the study protocol, enrolled volunteers were randomly assigned (stratified randomization based on body composition results) to the treatment order with specific codes by an impartial biostatistician. A 4-week washout period was introduced between treatments. The main study protocol included body mass and body composition evaluation, three saliva and blood samplings (resting [REST]; 3 min [POST-EX] and 60 min after completion of the second exercise protocol [REC]), and two exercise protocols during each of T_1_-T_4_ study visit (interspaced with 50 min of passive rest and 10 min of warm-up before the second exercise test). All testing was performed in the morning hours at the same time for the participant, to avoid physiological diurnal fluctuations in measured saliva and blood outcomes. The participants consumed a standardized meal three hours before the visits (20, 21) and the additional snack (two bananas) in between two exercise protocols.

#### 2.2.1 Supplementation

In the experimental procedure, each athlete was supplemented with a chronic (12 weeks) dose of 25 g·day^-1^ of COL and PLA treatment in a randomized crossover sequence. The supplement was particularly prepared for the study from a first post-delivery milking (up to 24 h post-delivery) and had a high content of IgG (30%; certified *Colostrum Bovinum*; Agrapak, Poland). PLA was an isoenergetic/isomacronutrient product (high-quality milk protein) prepared for the trial (Agrapak, Poland). The energy value of COL and PLA was about 357 kcal per 100 g; contained ≤1g of total fat and saturated fatty acids, 18 g carbohydrates, and ≤70 g total protein per 100 g of products. Regarding protein compounds characterized by biological activity COL preparation contained ≥38 g of IgG, ≥4.6 g proline rich peptides and ≥1 g lactoferrin per 100 g of product. The supplements were provided in powder form and were taken twice a day (12.5 g in the morning and 12.5 g in the afternoon). Participants were instructed to dissolve each portion of the supplement in 250 mL of plain water. The preparations were administrated to each participant in containers marked with a unique code. Under the recommended blinding procedure, the preparations were made in advance by the researcher who did not directly participate in the investigations. Regarding double-blinding, neither the researchers nor the participants knew whether COL or PLA was administered. Randomization details were anonymized and revealed after the protocol cessation.

#### 2.2.2 Body mass and body composition evaluation

All participants avoided strenuous exercise for at least 24 h prior to each visit. Anthropometric measurements were taken at the beginning of each study visit to ensure the same conditions for the testing procedures. Prior to body composition analysis, body mass, and height were measured in duplicate using a calibrated scale with a stadiometer (WPT 60/150 OW, Radwag, Poland). Analysis of body composition by electrical bioimpedance was conducted using a BIA-101ASE (Akern, Italy). During measurements, all the recommended procedures concerning measurement conditions were closely followed as described previously (22).

#### 2.2.3 Saliva and blood collection and sample analysis

Up to 10 minutes before REST and REC saliva sampling, and immediately before POST-EX saliva sampling, the mouth was rinsed with plain water for 1-5 s, as recommended by the manufacturer. To obtain the sample, the Salivette^®^ tubes (Sarstedt, Germany) were used. The participant removed the swab from the Salivette^®^ tube, placed the swab in the mouth and chewed it for about 60 s to stimulate salivation, then returned the swab to the Salivette^®^ tube. Saliva samples (swabs) were centrifuged for 2 min at 1000 g to allow the separation of the pellet and supernatant, and then storaged (at -80°C) for later analyses. Salivary SIgA was further analyzed using commercial ELISA kit (ref. 201-12-0197, SunRed, China) and read on the ELISA microplate reader Synergy 2 SIAFRT (BioTek Instruments, USA).

Participants remained seated, performing minimal movement for 10 min prior to each blood sampling, except for POST-EX samples, which were drawn 3 min after exercise cessation. Venous blood samples (~13 mL) were collected by venepuncture from an antecubital vein and separated immediately into the K2EDTA or clot activator vacutainers for the determination of biochemical markers. Analyses of WBC count and differential (lymphocytes, LYM; monocytes, MON; granulocytes, GRA) and hematological indices (red blood cells count, RBC; hemoglobin concentration, HGB; hematocrit value, HTC; mean corpuscular hemoglobin mass, MCH; mean corpuscular hemoglobin concentration, MCHC; mean corpuscular volume, MCV; mean platelet volume, MPV; platelet hematocrit, PCT; platelet distribution width, PDW; platelet count, PLT; platelet large cell ratio, PLCR) were analyzed immediately after blood sampling on hematology analyzer Mythic^®^ 18 (Orphée, Switzerland). Analyses of Igs (IgA, IgE, IgG, IgM), nutritional status indices (concentration of total protein, TP; albumin, ALB; urea, UREA; glucose, GLU), as well as muscle damage markers (concentration of creatinine, CREA; activity of alanine aminotransferase, ALT; aspartate aminotransferase, AST; creatine kinase, CK; lactate dehydrogenase, LDH) were performed from serum or plasma following blood samples centrifugation (4000 g for 10 min at 4°C) on the Accent 220S automatic biochemical analyzer (Cormay, Poland). Blood IgD was analyzed using a commercial ELISA kit (ref. 201-12-0175, SunRed, China) and read on the ELISA microplate reader Synergy 2 SIAFRT (BioTek Instruments, USA). In addition, to avoid potential misinterpretation of blood markers’ results, due to inter-individual variation in hydration status between study visits and measurements within the same study, hematology indices related to the number of blood cellular components (WBC, RBC, HGB, PLT) and blood biochemical parameters were converted using previously described hematocrit correction formula (20, 23–25).

#### 2.2.4 Exercise protocols

##### 2.2.4.1 Incremental rowing test

Two exercise protocols were implemented in this study. The first was performed immediately after the REST saliva/blood sampling and it was the incremental rowing test (IRT) to exhaustion. The test aimed at the evaluation of aerobic capacity. The test was performed on a rowing ergometer (Concept2, USA). The IRT started with a load of 50 W for 5 min (warm-up). Subsequently, the load was increased by 50 W every 3 min. The test continued until the subjective feeling of exhaustion of the athlete, i.e., refusal to undertake further physical exertion.

##### 2.2.4.2 Swimming-specific performance test

The second test was performed 60 min after the cessation of IRT (proceeded with 10-min warm-up in the indoor swimming pool). The test aimed at the evaluation of swimming-specific performance. The step test consisted of eight 100-meter-long sections to swim through, of which the sections I–III were performed at a level of 75% maximal effort [ME, determined during the familiarization visit], IV–V at 85% ME, VI at 90% ME, VII at 95% ME and VIII at 100% ME), with 1 to 2.5 min of recovery between sections. The test was a modification of the previously validated lactate threshold protocol (26, 27). The results of the exercise protocols are out of the scope of this manuscript.

#### 2.2.5 Statistical analysis

All variables were checked for a normal distribution with the Shapiro–Wilk test. Furthermore, kurtosis skewness (28), and a graphical evaluation of the distribution of each variable data were performed. Data transformation procedures (e.g., Box-Cox transformation) were not considered, while they did not result in obtaining normal distribution of all of the transformed variables. Baseline comparisons between subgroups according to the sequence of supplementation (PLA→COL *vs.* COL→PLA) were determined with the *T*-test for independent variables. Saliva and blood variables with a normal distribution were analyzed using a mixed model of analysis of variance with repeated measurements (RM ANOVA) with treatment sequence as a predictor (*treatment x sequence*), with the effect size (ES) expressed as partial eta-squared (*η^2p^*). A Huynh–Feldt adjustment was made when sphericity was violated (as indicated by Mauchly’s test). *Post-hoc* comparisons were performed by the Bonferroni test. If the normality assumptions were violated, the data were analyzed by Friedman’s ANOVA (ES expressed as Kendall’s concordance coefficient *W*) followed by the *post-hoc* for Friedman (based on average rank). Moreover, the possible carryover effect was evaluated based on the comparisons of REST measurements of all evaluated variables between visits T_1_ and T_3_. T_1_ *vs.* T_3_ differences were analyzed using *T*-test for dependent variables and ES expressed as Cohen’s *d* (normal distribution of the data) or the Wilcoxon signed-rank test, with the ES expressed as the rank correlation coefficient (*r_c_*) (data with non-normal distribution). An alpha of <0.05 was taken as a statistically significant value. The data were analyzed by using the STATISTICA 13.3 software (StatSoft Inc., USA).

## 3 Results

### 3.1 Saliva SIgA

**Figure 2:**
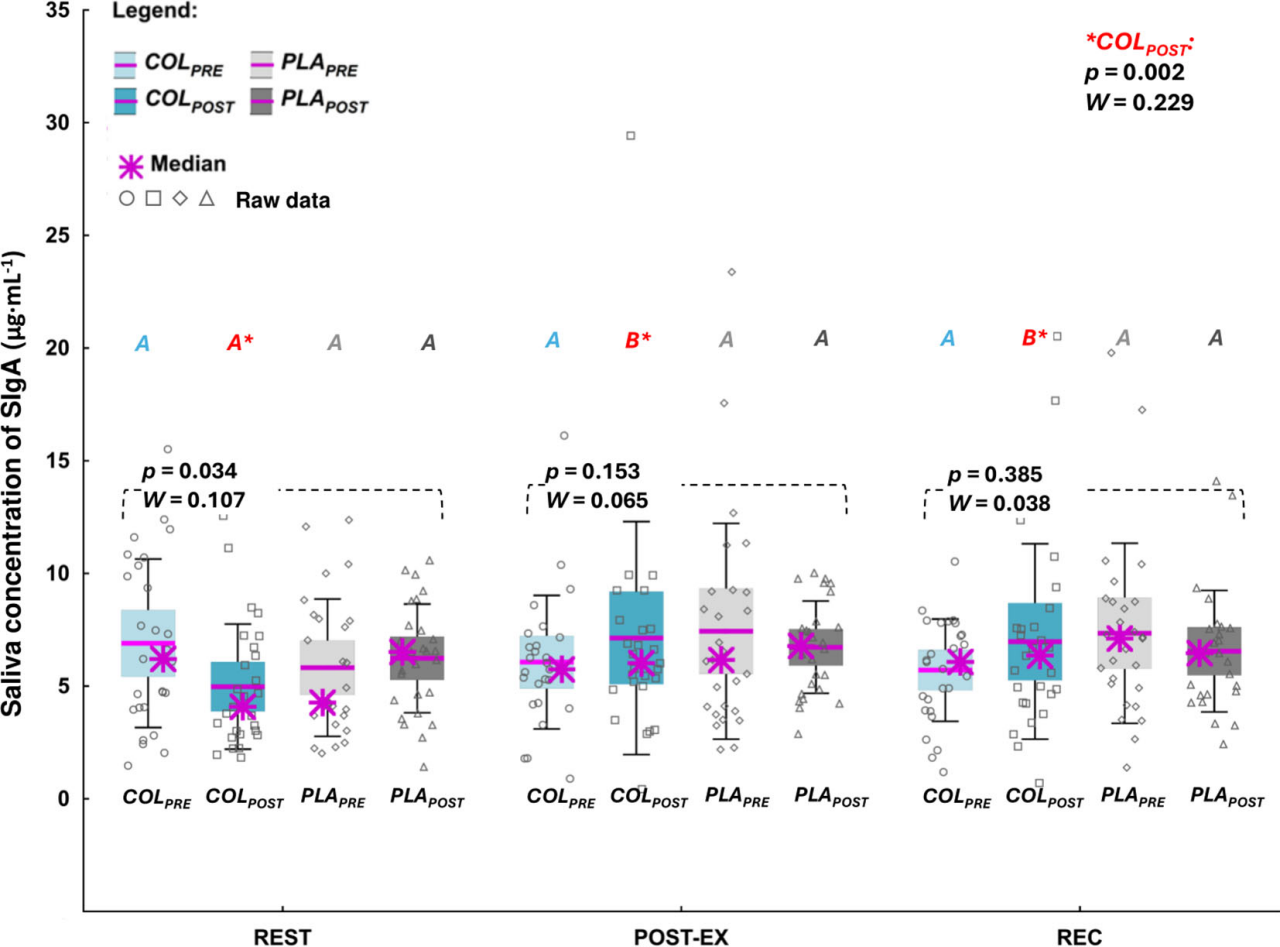
Saliva concentration of secretory immunoglobulin A (SIgA). The data are expressed as the median (asterisk), mean (line), 95% confidence interval (box), 95% CI + one standard deviation (whisker), and data of individuals. The data were analyzed with Friedman’s ANOVA followed by *post-hoc* for Friedman; the effect size is expressed as Kendall’s *W*. ^A,B^ different letters in red refer to significant differences between measuring time points (REST, POSTEX, and REC) during *COL_POST_* visit.

*Post-hoc* analysis did not indicate significant differences in REST, POST-EX, or REC concentrations of SIgA between the study visits. Nevertheless, at *COL_POST_*, SIgA concentration was significantly higher at POST-EX and REC compared to REST (*p*=0.002, *W*=0.229; **Figure 2**).

### 3.2 Blood Igs

**Figure 3:**
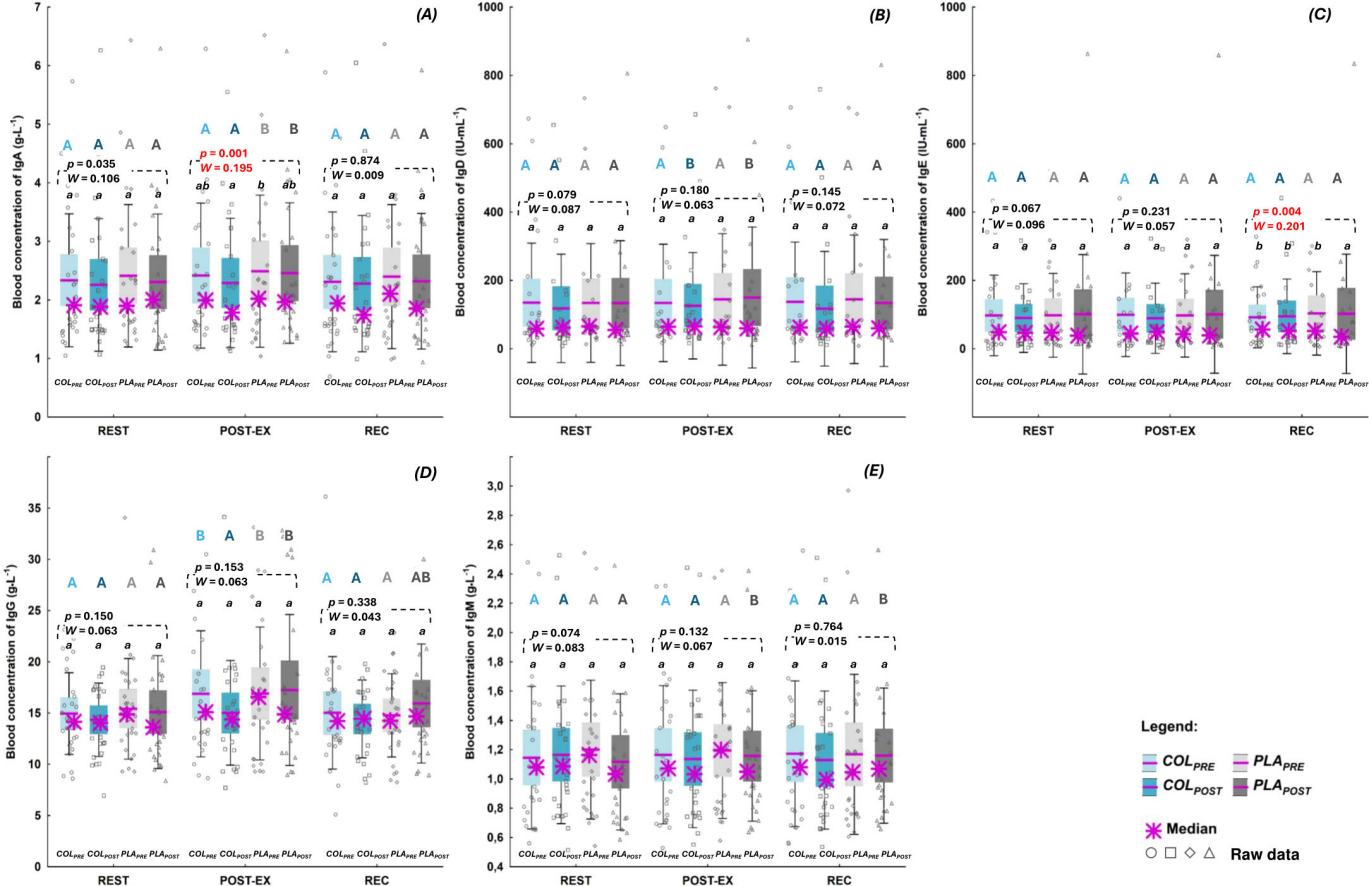
Blood concentration of: **(A)** immunoglobulin(Ig) A **(B)** IgD, **(C)** IgE, **(D)** IgG, and **(E)** IgM. The data are expressed as the median (asterisk), mean (line), 95% confidence interval (box), 95% CI + one standard deviation (whisker), and data of individuals. The data were analyzed with Friedman’s ANOVA followed by *post-hoc* for Friedman; the effect size is expressed as Kendall’s *W*. ^a,b^ different letters refer to significant differences between study visits (*COL_PRE_*, *COL_POST_*, *PLA_PRE_*, *PLA_POST_*). ^A,B^ different letters refer to significant differences between measuring time points (REST, POST-EX, and REC) within the same study visit (statistics for all significant differences [*p*, Kendall’s *W*] are given in the text).

Concentrations of blood Igs are presented in the **Figures 3A–E**. COL supplementation did not affect REST, POST-EX or REC concentration of measured blood Igs. The concentration of IgA at POST-EX was significantly lower at *COL_POST_* compared to *PLA_PRE_*, with no significant differences between the remaining study visits (*p*=0.001, *W*=0.195; **Figure 3A**). There were no differences in IgA concentrations between study visits at REST and REC time points. The concentrations of IgE at REC was significantly lower at *PLA_POST_* compared to the remaining study visits (*p*=0.004, *W*=0.201; **Figure 3C**). REST and POST-EX IgE concentrations did not differ between study visits (**Figure 3C**). Furthermore, there were no differences in blood concentrations of IgD (**Figure 3B**), IgG (**Figure 3D**), and IgM (**Figure 3E**) between any of the study visits at REST, POST-EX, or REC.

There were also no differences between *COL_POST_* and *PLA_POST_* concentrations of any of the studied blood Igs at none of the measuring time points (**Figures 3A–E**).

Apart from IgE (**Figure 3C**), there were significant differences in the concentrations of blood Igs between measuring time points (REST, POST-EX, and REC) within particular study visits. The concentrations of IgA at *PLA_PRE_* (*p*<0.000, *W*=0.365) and *PLA_POST_* (*p*<0.000, *W*=0.330; **Figure 3A**) were significantly higher at POST-EX compared to REST and REC. The concentrations of IgD at *COL_POST_* (*p*=0.001, *W*=0.286) and *PLA_POST_* (*p*=0.013, *W*=0.174; **Figure 3B**) were significantly higher at POST-EX compared to REST and REC. The concentrations of IgG *a)* at *COL_PRE_* (*p*<0.000, *W*=0.311) and *PLA_PRE_* (*p*=0.018, *W*=0.148) were significantly higher at POST-EX compared to REST and REC, *b)* at *PLA_POST_* (*p*=0.005, *W*=0.198; **Figure 3D**) were significantly higher at POST-EX compared to REST, while at *c) COL_POST_* there were no differences between measuring time points in IgG concentration. The concentration of IgM at *PLA_POST_* (*p*=0.002, *W*=0.240; **Figure 3E**) was significantly higher at POST-EX and REC compared to REST.

### 3.3 WBC count and differential

Regarding the simple effect of treatment, REST count of WBC was significantly higher only at the *COL_POST_* compared to the *PLA_PRE_* visit, with no differences between the remaining study visits (*p*=0.006, *η* ^2^ *_p_* =0.147; **Table 2**). POST-EX and REC counts of WBC did not differ between study visits. At REST count of LYM was significantly higher at *PLA_POST_* compared to *PLA_PRE_* (*p*=0.006; *W*=0.146), with no differences between the remaining visits. At REC count of LYM was significantly higher at *COL_POST_* and *PLA_POST_* visits compared to *PLA_PRE_* visit, with no differences between the remaining visits (*p*<0.000, *η* ^2^ *_p_* =0.240). There were no differences in POST-EX count of LYM between the study visits. MON and GRA counts did not differ across study visits at any of the measuring time points.

**Table 2:** White blood cell count and differential.

Indicator	Units	Measurement time point	*COL_PRE_*	*COL_POST_*	*PLA_PRE_*	*PLA_POST_*	*Treatment Sequence Treatment x sequence* [*p*]; *η* ^2^ *_p_* ^†^ or *Treatment* [*p*]; *W* ^§^
**WBC**	**(10^9^·L^-1^)**	**REST**	6.0 ± 1.3^ab/A^ (5.5 – 6.5)	6.5 ± 1.3^b/A^ (6.0 – 7.0)	5.6 ± 1.2^a/A^ (5.2 – 6.1)	6.3 ± 1.1^ab/A^ (5.9 – 6.7)	**[0.006]; 0.147^†^** [0.940]; 0.000**^†^** **[0.019]; 0.119^†^**
		**POST-EX**	10.4 ± 2.6^C^ (9.4 – 11.4)	10.6 ± 2.4^C^ (9.6 – 11.5)	10.3 ± 2.8^C^ (9.2 – 11.4)	10.3 ± 2.4^C^ (9.4 – 11.2)	[0.913]; 0.007**^†^** [0.659]; 0.008**^†^** **[0.035]; 0.104^†^**
		**REC**	7.8 ± 1.7^B^ (7.1 – 8.5)	8.3 ± 2.0^B^ (7.5 – 9.1)	7.9 ± 1.9^B^ (7.1 – 8.6)	7.9 ± 1.9^B^ (7.2 – 8.7)	[0.409]; 0.036**^†^** [0.435]; 0.024**^†^** **[0.024]; 0.113^†^**
		**[*p*]; *η* ^2^ *_p_* ^†^**	**[<0.000]; 0.708^†^**	**[<0.000]; 0.774^†^**	**[<0.000]; 0.728^†^**	**[<0.000]; 0.696^†^**	–
**LYM**	**(10^9^·L^-1^)**	**REST**	2.3 ± 0.5^ab/B^ (2.2 – 2.5)	2.5 ± 0.5^ab/B^ (2.3 – 2.7)	2.1 ± 1.0^a/B^ (2.0 – 2.3)	2.6 ± 0.5^b/B^ (2.4 – 2.8)	**[0.006]; 0.146^§^**
		**POST-EX**	3.7 ± 1.2^C^ (3.3 – 4.2)	3.8 ± 1.3^C^ (3.4 – 4.3)	3.6 ± 1.2^C^ (3.2 – 4.1)	3.9 ± 1.3^C^ (3.4 – 4.4)	[0.763]; 0.014^§^
		**REC**	1.7 ± 0.4^ab/A^ (1.6 – 1.9)	1.8 ± 0.4^b/A^ (1.7 – 1.9)	1.5 ± 0.4^a/A^ (1.4 – 1.7)	1.9 ± 0.4^b/A^ (1.7 – 2.0)	**[0.000]; 0.240^†^** [0.405]; 0.027^†^ **[<0.000]; 0.221^†^**
		**[*p*]; *η* ^2^ *_p_* ^†^ or *W* ^§^**	**[<0.000]; 1.000^§^**	**[<0.000]; 0.966^§^**	**[<0.000]; 0.738^†^**	**[<0.000]; 0.966^§^**	–
**MON**	**(10^9^·L^-1^)**	**REST**	0.4 ± 0.1^A^ (0.4 – 0.5)	0.4 ± 0.1^A^ (0.4 – 0.5)	0.4 ± 0.1^A^ (0.4 – 0.5)	0.5 ± 0.1^A^ (0.4 – 0.5)	[0.372]; 0.037^§^
		**POST-EX**	0.7 ± 0.2^B^ (0.6 – 0.7)	0.7 ± 0.2^B^ (0.6 – 0.8)	0.6 ± 0.2^B^ (0.6 – 0.7)	0.7 ± 0.2^B^ (0.6 – 0.7)	[0.778]; 0.014^†^ [0.743]; 0.004^†^ [0.094]; 0.078^†^
		**REC**	0.4 ± 0.1^A^ (0.4 – 0.5)	0.5 ± 0.2^A^ (0.4 – 0.5)	0.4 ± 0.1^A^ (0.3 – 0.4)	0.4 ± 0.1^A^ (0.4 – 0.5)	[0.253]; 0.049^§^
		**[*p*]; *η* ^2^ *_p_* ^†^ or *W* ^§^**	**[<0.000]; 0.668^§^**	**[<0.000]; 0.624^§^**	**[<0.0000]; 0.669^†^**	**[<0.000]; 0.552^§^**	–
**GRA**	**(10^9^·L^-1^)**	**REST**	3.2 ± 1.0^A^ (2.9 – 3.6)	3.5 ± 1.3^A^ (3.0 – 4.1)	3.1 ± 1.0^A^ (2.7 – 3.5)	3.3 ± 0.9^A^ (2.9 – 3.6)	[0.231]; 0.053^†^ [0.983]; 0.000^†^ [0.458]; 0.033^†^
		**POST-EX**	6.0 ± 2.0^B^ (5.3 – 6.8)	6.0 ± 2.3^B^ (5.1 – 6.9)	6.1 ± 2.3^B^ (5.2 – 7.0)	5.8 ± 2.0^B^ (5.0 – 6.5)	[0.846]; 0.010^§^
		**REC**	5.7 ± 1.7^B^ (5.0 – 6.3)	6.1 ± 2.2^B^ (5.2 – 6.9)	5.9 ± 1.8^B^ (5.2 – 6.7)	5.6 ± 1.8^B^ (4.9 – 6.7)	[0.354]; 0.039^§^
		**[*p*]; *η* ^2^ *_p_* ^†^ or *W* ^§^**	**[<0.000]; 0.655^†^**	**[<0.000]; 0.658** ^§^	**[<0.000]; 0.755** ^§^	**[<0.000]; 0.651^†^**	–
**LYM**	**(%)**	**REST**	39.6 ± 7.0^C^ (36.9 – 42.3)	39.7 ± 9.9^B^ (35.8 – 43.6)	39.2 ± 8.7^B^ (35.8 – 42.5)	41.6 ± 7.7^B^ (38.6 – 44.5)	[0.486]; 0.031^†^ [0.734]; 0.004^†^ [0.694]; 0.018^†^
		**POST-EX**	36.3 ± 8.9^B^ (32.8 – 39.7)	37.3 ± 10.9^B^ (33.1 – 41.5)	35.5 ± 9.2^B^ (32.0 – 39.1)	38.2 ± 10.3^B^ (34.2 – 42.2)	[0.455]; 0.031^§^
		**REC**	23.0 ± 6.9^ab/A^ (20.3 – 25.7)	22.9 ± 7.4^ab/A^ (20.1 – 25.8)	20.5 ± 6.3^a/A^ (18.1 – 23.0)	24.5 ± 6.9^b/A^ (21.8 – 27.2)	**[0.001]; 0.194** ^§^
		**[*p*]; *η* ^2^ *_p_* ^†^ or *W* ^§^**	**[<0.000]; 0.800^†^**	**[<0.000]; 0.766^†^**	**[<0.000]; 0.761** ^§^	**[<0.000]; 0.761** ^§^	–
**MON**	**(%)**	**REST**	7.3 ± 2.0^C^ (6.5 – 8.0)	6.9 ± 2.3^B^ (6.0 – 7.8)	7.2 ± 2.0^C^ (6.5 – 8.0)	7.4 ± 2.1^C^ (6.6 – 8.2)	[0.356]; 0.039^§^
		**POST-EX**	6.5 ± 1.8^B^ (5.8 – 7.2)	6.6 ± 2.2^B^ (5.7 – 7.5)	6.2 ± 1.7^B^ (5.6 – 6.9)	6.7 ± 2.0^B^ (6.0 – 7.5)	[0.953]; 0.004^§^
		**REC**	5.7 ± 1.8^A^ (5.0 – 6.4)	5.9 ± 2.9^A^ (4.8 – 7.1)	5.2 ± 1.5^A^ (4.6 – 5.7)	5.8 ± 2.3^A^ (4.9 – 6.7)	[0.719]; 0.016^§^
		**[*p*]; *η* ^2^ *_p_* ^†^ or *W* ^§^**	**[<0.000]; 0.570^†^**	**[<0.000]; 0.331** ^§^	**[<0.000]; 0.615^†^**	**[<0.000]; 0.557** ^§^	–
**GRA**	**(%)**	**REST**	53.2 ± 7.5^A^ (50.2 – 56.1)	53.4 ± 10.6^A^ (49.3 – 57.5)	53.6 ± 9.0^A^ (50.1 – 57.1)	51.0 ± 8.2^A^ (47.8 – 54.2)	[0.442]; 0.034^†^ [0.781]; 0.003^†^ [0.967]; 0.003^†^
		**POST-EX**	57.2 ± 9.9^B^ (53.4 – 61.0)	56.1 ± 11.7^A^ (51.5 – 60.6)	58.2 ± 10.0^B^ (54.3 – 62.1)	55.1 ± 11.3^A^ (50.7 – 59.5)	[0.113]; 0.073^†^ [0.529]; 0.015^†^ [0.210]; 0.056^†^
		**REC**	71.3 ± 8.0^ab/C^ (68.2 – 74.4)	71.1 ± 8.8^ab/B^ (67.6 – 74.5)	74.3 ± 7.0^b/C^ (71.6 – 77.0)	69.2 ± 8.1^a/B^ (66.0 – 72.3)	**[0.007]; 0.145** ^§^
		**[*p*]; *η* ^2^ *_p_* ^†^ or *W* ^§^**	**[<0.000]; 0.810^†^**	**[<0.000]; 0.773^†^**	**[<0.000]; 0.826^†^**	**[<0.000]; 0.770** ^§^	–

The results are expressed as the mean ± standard deviation and 95% confidence interval (in parentheses). GRA, granulocytes; LYM, lymphocytes; MON, monocytes; WBC, white blood cells. ^†^The data were analyzed with a mixed model of RM ANOVA with treatment sequence as a predictor (*treatment x sequence*) followed by the Bonferroni test; the effect size is expressed as partial eta-squared (η^2^ *_p_*). ^§^The data were analyzed with Friedman’s ANOVA followed by *post-hoc* for Friedman; the effect size is expressed as Kendall’s *W*. ^a,b^different letters refer to significant differences between study visits for simple effect of *treatment* (*COL_PRE_, COL_POST_, PLA_PRE_, PLA_POST_*). ^A,B,C^different letters refer to significant differences between measuring time points (REST, POST-EX, and REC) within the same study visit.Results in bold refer to statistically significant differences.

There were no differences in WBC, LYM, MON, or GRA at any of the measuring time point between *COL_POST_* and *PLA_POST_* (**Table 2**).

There were significant *treatment x sequence* interactions for: *a)* WBC at REST (*p*=0.019, *η* ^2^ *_p_* =0.119), POST-EX (*p*=0.035, *η* ^2^ *_p_* =0.104; *post-hoc* did not indicate differences), and REC (*p*=0.024, *η* ^2^ *_p_* =0.113; *post-hoc* did not indicate differences); and *b)* LYM at REC (*p*<0.000, *η* ^2^ *_p_* =0.221).

The percentage of LYM at REC was significantly higher at *PLA_POST_* compared to *PLA_PRE_*, with no differences between the remaining study visits (*p*=0.001, *W*=0.194; **Table 2**). While, the percentage of GRA at REC was significantly lower at *PLA_POST_* compared to *PLA_PRE_*, with no differences between the remaining study visits (*p*=0.007, *W*=0.145). The percentage of LYM and GRA at REST and POST-EX did not differ between the study visits. The percentage of MON was unchanged across study visits and measuring time points (**Table 2**).

The percentage of LYM, MON, and GRA did not differ between *COL_POST_* and *PLA_POST_* at any of the measuring time point (**Table 2**).

There were exercise-induced increases in total WBC count and differential across all study visits (**Table 2**). Regardless of the study visit, the WBC count increased significantly from REST to POST-EX, and at REC it was significantly lower compared to POST-EX, but still higher compared to REST (did not return to baseline level). Regardless of the study visit, LYM counts increased significantly from REST to POST-EX, and dropped from POST-EX to REC to the level compared to REST. The count of MON was significantly higher at POST-EX compared to REST and REC, with no differences between REST and REC. The count of GRA was significantly higher at POST-EX and REC compared to REST, with no differences between POST-EX and REC. There were exercise-induced variations in the percentage of WBC differential between measuring time points (REST *vs*. POST-EX *vs*. REC) within the same visit, but still the variations were visit-specific and they are indicated in **Table 2** by uppercase latter superscripts.

### 3.4 Blood hematological indices

Regarding the simple effect of *treatment*, RBC count at REST was significantly lower at *PLA_POST_* compared to *PLA_PRE_*, with no differences between remaining study visits (*p*=0.048, *η* ^2^ *_p_* =0.096; **Table 3**). There were no differences in RBC at POST-EX and REC between study visits. HTC at REC was significantly lower at *COL_POST_* compared to *PLA_POST_*, with no differences between remaining study visits (*p*=0.024, *W*=0.116). There were no differences in HTC at REST and POST-EX between study visits. There were no differences in other hematological indices (HGB, MCV, MCH, MCHC, RDW-C, and RDW-S) between study visits at any of the measuring time points (**Table 3**).

**Table 3:** Red blood cell- and platelet-related indices.

Indicator	Units	Measurement time point	*COL_PRE_*	*COL_POST_*	*PLA_PRE_*	*PLA_POST_*	*Treatment Sequence Treatment x sequence* [*p*]; *η* ^2^ *_p_* ^†^ or *Treatment* [*p*]; *W* ^§^
**RBC**	**(10^12^·L^-1^)**	**REST**	5.80 ± 0.25^ab/B^ (5.71 – 5.90)	5.79 ± 0.22^ab/B^ (5.70 – 5.88)	5.84 ± 0.24^b/B^ (5.75 – 5.93)	5.76 ± 0.23^a/B^ (5.67 – 5.85)	**[0.048]; 0.096** ^†^ [0.249]; 0.051^†^ **[<0.000]; 0.266** ^†^
		**POST-EX**	5.76 ± 0.24^A^ (5.67 – 5.85)	5.73 ± 0.22^A^ (5.65 – 5.82)	5.78 ± 0.22^A^ (5.69 – 5.86)	5.73 ± 0.23^A^ (5.64 – 5.82)	[0.329]; 0.043^†^ [0.213]; 0.059^†^ **[0.001]; 0.202** ^†^
		**REC**	5.82 ± 0.25^B^ (5.72 – 5.91)	5.78 ± 0.23^B^ (5.70 – 5.87)	5.83 ± 0.24^B^ (5.73 – 5.92)	5.77 ± 0.22^B^ (5.69 – 5.85)	[0.179]; 0.061^†^ [0.340]; 0.035^†^ **[0.006]; 0.148** ^†^
		**[*p*]; *η* ^2^ *_p_* ^†^**	**[<0.000]; 0.439^†^**	**[<0.000]; 0.444^†^**	**[<0.000]; 0.277^†^**	**[<0.000]; 0.255^†^**	–
**HGB**	**(mmol·L^-1^)**	**REST**	10.63 ± 0.53^B^ (10.43 – 10.84)	10.74 ± 0.49^B^ (10.55 – 10.93)	10.58 ± 0.38^B^ (10.43 – 10.72)	10.71 ± 0.38^B^ (10.57 – 10.86)	[0.292]; 0.044^§^
		**POST-EX**	10.49 ± 0.52^A^ (10.29 – 10.69)	10.62 ± 0.45^A^ (10.45 – 10.79)	10.45 ± 0.32^A^ (10.33 – 10.57)	10.59 ± 0.34^A^ (10.46 – 10.72)	**[0.049]; 0.093** ^§^
		**REC**	10.61 ± 0.49^B^ (10.42 – 10.80)	10.66 ± 0.45^A^ (10.49 – 10.84)	10.59 ± 0.31^B^ (10.47 – 10.71)	10.73 ± 0.36^B^ (10.59 – 10.87)	[0.201]; 0.055^§^
		**[*p*]; *η* ^2^ *_p_* ^†^ or *W* ^§^**	**[0.001]; 0.246** ^§^	**[0.001]; 0.224^†^**	**[<0.000]; 0.352^†^**	**[<0.000]; 0.336^†^**	–
**HTC**	**(L·L^-1^)**	**REST**	0.434 ± 0.027^B^ (0.423 – 0.444)	0.438 ± 0.025^B^ (0.428 – 0.447)	0.435 ± 0.028^B^ (0.424 – 0.446)	0.435 ± 0.026^A^ (0.425 – 0.445)	[0.819]; 0.011^§^
		**POST-EX**	0.454 ± 0.041^C^ (0.438 – 0.469)	0.456 ± 0.029^C^ (0.445 – 0.467)	0.458 ± 0.034^C^ (0.445 – 0.471)	0.458 – 0.028^B^ (0.447 – 0.468)	[0.808]; 0.012^†^ [0.874]; 0.001^†^ **[0.017]; 0.121** ^†^
		**REC**	0.425 ± 0.038^ab/A^ (0.411 – 0.440)	0.423 ± 0.027^a/A^ (0.412 – 0.433)	0.425 ± 0.032^ab/A^ (0.413 – 0.437)	0.426 ± 0.029^b/A^ (0.415 – 0.437)	**[0.024]; 0.116** ^§^
		**[*p*]; *η* ^2^ *_p_* ^†^ or *W* ^§^**	**[<0.000]; 0.855** ^§^	**[<0.000]; 0.829^†^**	**[<0.000]; 0.876** ^§^	**[<0.000]; 0.566^†^**	–
**MCV**	**(fL)**	**REST**	86.3 ± 3.6^A^ (84.9 – 87.7)	86.5 ± 3.4^A^ (85.2 – 87.8)	85.8 ± 3.5^A^ (84.4 – 87.1)	87.0 ± 3.6^A^ (85.5 – 88.4)	[0.057]; 0.095**^†^** [0.226]; 0.058**^†^** **[<0.000]; 0.267^†^**
		**POST-EX**	87.0 ± 3.6^B^ (85.6 – 88.3)	87.3 ± 3.4^B^ (86.0 – 88.6)	86.7 ± 3.3^B^ (85.4 – 88.0)	87.4 ± 3.4^B^ (86.0 – 88.7)	[0.331]; 0.043^†^ [0.190]; 0.065^†^ **[<0.000]; 0.206** ^†^
		**REC**	86.1 ± 3.6^A^ (84.7 ± 87.5)	86.6 ± 3.4^A^ (85.2 – 87.9)	85.9 ± 3.5^A^ (84.5 – 87.3)	86.8 ± 3.3^A^ (85.5 – 88.1)	[0.282]; 0.049^†^ [0.283]; 0.046^†^ **[0.004]; 0.160** ^†^
		**[*p*]; *η* ^2^ *_p_* ^†^**	**[<0.000]; 0.438^†^**	**[<0.000]; 0.447^†^**	**[<0.000]; 0.270^†^**	**[<0.000]; 0.377^†^**	–
**MCH**	**(fmol)**	**REST**	1.83 ± 0.12^B^ (1.79 – 1.88)	1.86 ± 0.11^B^ (1.82 – 1.90)	1.81 ± 0.08 (1.78 – 1.84)	1.86 ± 0.12 (1.82 – 1.91)	[0.078]; 0.083^†^ [0.484]; 0.019^†^ **[0.021]; 0.116** ^†^
		**POST-EX**	1.82 ± 0.12^A^ (1.78 – 1.87)	1.85 ± 0.11^AB^ (1.81 – 1.90)	1.81 ± 0.08 (1.78 – 1.84)	1.85 ± 0.11 (1.81 – 1.89)	[0.104]; 0.076^†^ [0.395]; 0.028^†^ **[0.004]; 0.155** ^†^
		**REC**	1.83 ± 0.12^AB^ (1.78 – 1.87)	1.85 ± 0.10^A^ (1.81 – 1.89)	1.82 ± 0.08 (1.79 – 1.85)	1.86 ± 0.11 (1.82 – 1.91)	[0.141]; 0.070^†^ [0.556]; 0.014^†^ **[0.012]; 0.135** ^†^
		**[*p*]; *η* ^2^ *_p_* ^†^**	**[0.041]; 0.112^†^**	**[0.040]; 0.112^†^**	[0.433]; 0.029**^†^**	[0.090]; 0.088**^†^**	–
**MCHC**	**(mmol·L^-1^)**	**REST**	21.27 ± 1.05^B^ (20.86 – 21.68)	21.48 ± 0.97^B^ (21.11 – 21.86)	21.16 ± 0.75^B^ (20.87 – 21.45)	21.43 ± 0.75^B^ (21.14 – 21.73)	[0.281]; 0.045^§^
		**POST-EX**	20.99 ± 1.04^A^ (20.58 – 21.39)	21.23 ± 0.89^A^ (20.89 – 21.58)	20.90 ± 0.63^A^ (20.65 – 21.14)	21.17 ± 0.67^A^ (20.91 – 21.44)	[0.059]; 0.088^§^
		**REC**	21.21 ± 0.99^B^ (20.83 – 21.59)	21.32 ± 0.91^A^ (20.97 – 21.67)	21.18 ± 0.62^B^ (20.94 – 21.43)	21.46 ± 0.73^B^ (21.17 – 21.75)	[0.138]; 0.068^§^
		**[*p*]; *η* ^2^ *_p_* ^†^ or *W* ^§^**	**[0.001]; 0.235^§^**	**[0.001]; 0.223^†^**	**[<0.000]; 0.384^§^**	**[<0.000]; 0.333^†^**	–
**RDW-C**	**(%)**	**REST**	12.1 ± 0.9 (11.8 – 12.5)	12.3 ± 1.1 (11.9 – 12.7)	12.4 ± 1.0 (12.0 – 12.8)	12.1 ± 0.9 (11.7 – 12.4)	[0.607]; 0.023^†^ [0.644]; 0.008^†^ [0.075]; 0.084^†^
		**POST-EX**	12.0 ± 0.9 (11.7 – 12.4)	12.3 ± 0.9 (11.9 – 12.7)	12.2 ± 0.9 (11.9 – 12.6)	12.1 ± 0.8 (11.8 – 12.4)	[0.385]; 0.038^§^
		**REC**	12.2 ± 1.0 (11.9 – 12.6)	12.4 ± 1.0 (12.0 – 12.8)	12.5 ± 1.0 (12.1 – 12.9)	12.2 ± 1.0 (11.8 – 12.6)	[0.601]; 0.024**^†^** [0.653]; 0.008^†^ [0.064]; 0.092^†^
		**[*p*]; *η* ^2^ *_p_* ^†^ or *W* ^§^**	[0.057]; 0.100**^†^**	[0.553]; 0.021^§^	[0.083]; 0.088^†^	[0.199]; 0.063^†^	–
**RDW-S**	**(fL)**	**REST**	40.2 ± 2.8^A^ (39.2 – 41.3)	41.3 ± 3.3^A^ (40.0 – 42.5)	40.9 ± 3.8 (39.4 – 42.4)	41.8 ± 3.8 (40.4 – 43.3)	[0.151]; 0.067^†^ [0.355]; 0.033^†^ **[<0.000]; 0.276** ^†^
		**POST-EX**	41.1 ± 2.7^B^ (40.0 – 42.1)	42.0 ± 3.3^B^ (40.7 – 43.3)	41.0 ± 3.8 (39.5 – 42.5)	42.3 ± 4.0 (40.8 – 43.9)	[0.183]; 0.061^†^ [0.222]; 0.057^†^ **[<0.000]; 0.325** ^†^
		**REC**	40.1 ± 2.8^B^ (40.0 – 42.1)	41.7 ± 2.9^AB^ (40.6 – 42.9)	40.9 ± 3.8 (39.4 – 42.3)	42.2 ± 3.6 (40.8 – 43.7)	[0.194]; 0.061^†^ [0.338]; 0.037^†^ **[<0.000]; 0.315** ^†^
		**[*p*]; *η* ^2^ *_p_* ^†^**	**[0.010]; 0.156^†^**	**[0.031]; 0.121** ^†^	[0.868]; 0.005**^†^**	[0.349]; 0.040**^†^**	–
**PLT**	**(10^9^·L^-1^)**	**REST**	272 ± 68^A^ (245 – 298)	272 ± 76^A^ (242 – 301)	276 ± 90^A^ (241 – 311)	276 ± 79^A^ (245 – 307)	[0.929]; 0.006^†^ [0.143]; 0.081^†^ [0.244]; 0.052^†^
		**POST-EX**	315 ± 70^B^ (288 – 342)	324 ± 82^C^ (292 – 356)	325 ± 108^B^ (283 – 366)	320 ± 85^B^ (287 – 353)	[0.905]; 0.007^†^ [0.282]; 0.044^†^ [0.317]; 0.043^†^
		**REC**	272 ± 70^A^ (245 – 299)	290 ± 77^B^ (260 – 320)	278 ± 96^A^ (241 – 316)	283 ± 76^A^ (253 – 312)	[0.644]; 0.021^†^ [0.258]; 0.049^†^ [0.465]; 0.030^†^
		**[*p*]; *η* ^2^ *_p_* ^†^**	**[<0.000]; 0.352^†^**	**[<0.000]; 0.661^†^**	**[<0.000]; 0.475^†^**	**[<0.000]; 0.607^†^**	–
**MPV**	**(fL)**	**REST**	8.7 ± 0.8^B^ (8.5 – 9.0)	8.7 ± 0.7^B^ (8.4 – 9.0)	8.7 ± 0.8^B^ (8.4 – 9.0)	8.7 ± 0.8^B^ (8.4 – 9.0)	[0.677]; 0.019^†^ [0.171]; 0.071^†^ **[0.048]; 0.096** ^†^
		**POST-EX**	8.8 ± 0.9^B^ (8.4 – 9.1)	8.7 ± 0.8^B^ (8.4 – 9.0)	8.8 ± 0.8^B^ (8.5 – 9.1)	8.9 ± 0.8^C^ (8.6 – 9.1)	[0.284]; 0.047^†^ [0.194]; 0.064^†^ [0.556]; 0.026^†^
		**REC**	8.5 ± 0.8^A^ (8.2 – 8.8)	8.3 ± 0.8^A^ (8.0 – 8.6)	8.3 ± 0.7^A^ (8.0 – 8.6)	8.4 ± 0.8^A^ (8.1 – 8.7)	[0.235]; 0.055^†^ [0.328]; 0.038^†^ [0.652]; 0.091^†^
		**[*p*]; *η* ^2^ *_p_* ^†^**	**[<0.000]; 0.231^†^**	**[<0.000]; 0.477^†^**	**[<0.000]; 0.341^†^**	**[<0.000]; 0.428^†^**	–
**PCT**	**(cl·L^-1^)**	**REST**	0.203 ± 0.045^A^ (0.186 – 0.220)	0.203 ± 0.047^A^ (0.185 – 0.222)	0.205 ± 0.058^A^ (0.183 – 0.228)	0.205 ± 0.054^A^ (0.184 – 0.226)	[0.817]; 0.011**^§^**
		**POST-EX**	0.246 ± 0.049^B^ (0.227 – 0.265)	0.253 ± 0.056^B^ (0.231 – 0.274)	0.256 ± 0.077^B^ (0.226 – 0.286)	0.257 ± 0.068^B^ (0.231 – 0.283)	[0.303]; 0.043^§^
		**REC**	0.195 ± 0.050^A^ (0.176 – 0.215)	0.200 ± 0.046^A^ (0.182 – 0.218)	0.195 ± 0.063^A^ (0.170 – 0.219)	0.199 ± 0.050^A^ (0.180 – 0.219)	[0.851]; 0.010**^§^**
		**[*p*]; *η* ^2^ *_p_* ^†^ or *W* ^§^**	**[<0.000]; 0.642^†^**	**[<0.000]; 0.765^§^**	**[<0.000]; 0.706^†^**	**[<0.000]; 0.7690** ^§^	–
**PDW**	**(%)**	**REST**	13.8 ± 1.3 (13.3 – 14.3)	13.7 ± 1.7 (13.1 – 14.3)	13.8 ± 1.6 (13.2 – 14.4)	14.1 ± 1.4 (13.5 – 14.6)	[0.778]; 0.014^†^ [0.642]; 0.008^†^ [0.718]; 0.017^†^
		**POST-EX**	14.0 ± 1.2 (13.6 – 14.5)	14.2 ± 1.3 (13.7 – 14.7)	14.7 ± 1.4 (14.2 – 15.2)	14.2 ± 1.1 (13.8 – 14.6)	[0.158]; 0.064^†^ [0.152]; 0.077^†^ [0.763]; 0.015^†^
		**REC**	13.9 ± 1.4 (13.3 – 14.4)	14.1 ± 1.4 (13.6 – 14.7)	14.3 ± 1.1 (13.8 – 14.7)	13.7 ± 1.1 (13.2 – 14.1)	[0.137]; 0.073**^†^** [0.931]; 0.000^†^ [0.620]; 0.024^†^
		**[*p*]; *η* ^2^ *_p_* ^†^**	[0.786]; 0.009**^†^**	[0.322]; 0.041**^†^**	[0.091]; 0.088**^†^**	[0.277]; 0.048**^†^**	–
**PLCR**	**(%)**	**REST**	19.0 ± 7.0^ab/B^ (16.3 – 21.7)	16.0 ± 4.6^ab/B^ (14.2 – 17.7)	19.4 ± 8.9^b/A^ (15.9 0 22.9)	15.4 ± 4.9^a/A^ (13.6 – 17.3)	**[0.014]; 0.126^§^**
		**POST-EX**	18.8 ± 6.9^AB^ (16.1 – 21.5)	15.5 ± 5.1^B^ (13.6 – 17.5)	19.6 ± 8.8^A^ (16.2 – 23.0)	16.7 ± 5.3^B^ (14.6 – 18.7)	[0.069]; 0.085**^§^**
		**REC**	17.5 ± 6.4^A^ (15.0 – 20.0_	13.7 ± 4.9^A^ (11.8 – 15.6)	16.2 ± 6.4^A^ (13.7 – 18.6)	13.8 ± 4.9^A^ (11.9 – 15.7)	**[0.014]; 0.131^§^**
		**[*p*]; *η* ^2^ *_p_* ^†^ or *W* ^§^**	**[0.034]; 0.118^†^**	**[0.002]; 0.218^§^**	**[0.010]; 0.164^§^**	**[<0.000]; 0.374^†^**	–

The results are expressed as the mean ± standard deviation and 95% confidence interval (in parentheses). HTC, hematocrit; HGB, hemoglobin; MCH, mean corpuscular hemoglobin mass; MCHC, mean corpuscular hemoglobin concentration; MCV, mean corpuscular volume; MPV, mean platelet volume; PCT, platelet hematocrit; PDW, platelet distribution width; PLT, platelet count; PLCR, platelet large cell ratio; RBC, red blood cells; RDW-C, red blood cells distribution width – coefficient of variation; RDW-S, red blood cells distribution width – standard deviation. ^†^The data were analyzed with a mixed model of RM ANOVA with treatment sequence as a predictor (*treatment x sequence*) followed by the Bonferroni test; the effect size is expressed as partial eta-squared (η^2^ *_p_*). ^§^The data were analyzed with Friedman’s ANOVA followed by *post-hoc* for Friedman; the effect size is expressed as Kendall’s *W*. ^a,b^different letters refer to significant differences between study visits for simple effect of *treatment* (*COL_PRE_, COL_POST_, PLA_PRE_, PLA_POST_*). ^A,B,C^different letters refer to significant differences between measuring time points (REST, POST-EX, and REC) within the same study visit.Results in bold refer to statistically significant differences.

Still, there were significant *treatment x sequence* interactions for: *a)* RBC at REST (*p*<0.000, *η* ^2^ *_p_* =0.266), POST-EX (*p*=0.001, *η* ^2^ *_p_* =0.202), and REC (*p*=0.006, *η* ^2^ *_p_* =0.148; *post-hoc* did not indicate differences); *b)* HTC at POST-EX (*p*=0.017, *η* ^2^ *_p_* =0.121; *post-hoc* did not indicate differences); *c)* MCV at REST (*p*<0.000, *η* ^2^ *_p_* =0.267), POST-EX (*p*<0.000, *η* ^2^ *_p_* =0.206) and REC (*p*=0.004, *η* ^2^ *_p_* =0.160); *d)* MCH at REST (*p*=0.021, *η* ^2^ *_p_* =0.116; *post-hoc* did not indicate differences), POST-EX (*p*=0.004, *η* ^2^ *_p_* =0.155) and REC (*p*=0.012, *η* ^2^ *_p_* =0.135; *post-hoc* did not indicate differences); *e)* RDW-S at REST (*p*<0.000, *η* ^2^ *_p_* =0.276); POST-EX (*p*<0.000, *η* ^2^ *_p_* =0.325) and REC (*p*<0.000, *η* ^2^ *_p_* =0.315).

Regarding the simple effect of *treatment*, there were no differences in PLT, MPV, PCT and PDW between study visits at any of the measuring time points (**Table 3**). Solely, PLCR at REST differed significantly between study visits, being significantly lower at *PLA_POST_* compared to *PLA_PRE_*, with no differences between study visits (*p*=0.014, *W*=0.126; **Table 3**). There were no differences in PLCR at POST-EX and REC between study visits.

There was a significant *treatment x sequence* interaction for MPV at REST (*p*<0.048, *η* ^2^ *_p_* =0.096; *post-hoc* did not indicate differences). Furthermore, there were exercise-induced variations in RBC- and PLT-related indices between measuring time points (REST *vs*. POST-EX *vs*. REC) within the same visit and they are indicated in **Table 3** by uppercase latter superscripts.

### 3.5 Nutritional status and muscle damage indices in blood

There were no differences in the activity of ALT, AST, CK and LDH between study visits at any of measuring time points (**Table 4**). Similarly, there were no differences in concentrations of TP, ALB, CREA, UREA, and GLU between study visits at any of measuring time points (**Table 4**).

**Table 4:** Muscle damage and nutritional status indices in blood.

Indicator	Units	Measurement time points	*COL_PRE_*	*COL_POST_*	*PLA_PRE_*	*PLA_POST_*	*Treatment Sequence Treatment x sequence* [*p*]; *η* ^2^ *_p_* ^†^ or *Treatment* [*p*]; *W* ^§^
**Alanine aminotransferase**	**(U·L^-1^)**	**REST**	25.7 ± 7.6^A^ (23.1 – 30.6)	25.6 ± 15.6^A^ (20.3 – 35.8)	27.1 ± 11.8^A^ (21.6 – 33.4)	31.0 ± 14.2^A^ (22.9 – 37.1)	[0.182]; 0.060**^§^**
		**POST-EX**	30.2 ± 8.5^B^ (27.4 – 35.9)	29.3 ± 16.6^C^ (23.9 – 40.5)	32.3 ± 9.3^B^ (26.4 – 35.7)	34.1 ± 13.1^B^ (28.3 – 41.4)	[0.245]; 0.050**^§^**
		**REC**	26.4 ± 8.6^A^ (24.7 – 33.3)	26.3 ± 15.2^B^ (21.5 – 36.7)	27.7 ± 10.2^A^ (22.8 – 33.0)	31.9 ± 14.1^A^ (24.0 – 38.1)	[0.413]; 0.035**^§^**
		**[*p*]; *W* ^§^**	**[<0.000]; 0.685^§^**	**[<0.000]; 0.684^§^**	**[<0.000]; 0.563^§^**	**[<0.000]; 0.701^§^**	–
**Asparagine aminotramsferase**	**(U·L^-1^)**	**REST**	28.2 ± 10.2^A^ (24.6 – 34.8)	26.6 ± 19.6^A^ (21.9 – 41.5)	29.6 ± 8.7^A^ (23.6 – 32.3)	30.6 ± 12.1^A^ (23.7 – 35.7)	[0.960]; 0.004**^§^**
		**POST-EX**	34.3 ± 9.0^C^ (30.9 – 39.9)	32.0 ± 20.1^C^ (26.1 – 46.2)	33.1 ± 10.8^C^ (28.0 – 38.7)	34.2 ± 12.2^C^ (28.8 – 41.0)	[1.000]; 0.000**^§^**
		**REC**	31.5 ± 11.2^B^ (26.3 – 37.4)	27.4 ± 20.3^B^ (24.0 – 44.3)	31.3 ± 13.9^B^ (24.2 – 38.1)	32.3 ± 15.6^B^ (26.0 – 41.6)	[0.997]; 0.000**^§^**
		**[*p*]; *W* ^§^**	**[<0.000]; 0.695^§^**	**[<0.000]; 0.801^§^**	**[<0.000]; 0.523^§^**	**[<0.000]; 0.679^§^**	–
**Creatine kinase**	**(U·L^-1^)**	**REST**	246.2 ± 136.3^A^ (197.9 – 334.2)	225.8 ± 201.0^A^ (157.3 – 358.3)	254.7 ± 159.5^A^ (150.6 – 310.0)	195.3 ± 186.7^A^ (165.4 – 352.1)	[0.372]; 0.037**^§^**
		**POST-EX**	297.9 ± 194.2^B^ (234.5 – 428.7)	265.3 ± 230.3^B^ (184.9 – 415.2)	301.1 ± 187.6^B^ (187.3 – 374.9)	243.2 ± 201.9^B^ (200.3 – 402.2)	[0.346]; 0.041**^§^**
		**REC**	306.7 ± 207.1^B^ (215.1 – 422.2)	292.9 ± 275.1^B^ (182.6 – 457.7)	299.6 ± 202.3^B^ (185.2 – 387.5)	263.2 ± 216.8^B^ (200.2 – 417.0)	[0.529]; 0.028**^§^**
		**[*p*]; *W* ^§^**	**[<0.000]; 0.778^§^**	**[<0.000]; 0.778^§^**	**[<0.000]; 0.632^§^**	**[<0.000]; 0.736^§^**	–
**Lactate** **dehydrogenase**	**(U·L^-1^)**	**REST**	422 ± 98^A^ (381 – 479)	402 ± 100^A^ (358 – 458)	406 ± 94^A^ (364 – 458)	426 ± 99^A^ (373 – 472)	[0.960]; 0.003**^§^**
		**POST-EX**	483 ± 110^B^ (422 – 532)	469 ± 93^C^ (413 – 506)	453 ± 110^B^ (409 – 520)	463 ± 92^B^ (429 – 520)	[0.540]; 0.027**^§^**
		**REC**	465 ± 117^B^ (418 – 536)	431 ± 93^B^ (402 – 495)	437 ± 126^B^ (402 – 528)	443 ± 78^A^ (406 – 483)	[0.698]; 0.018**^§^**
		**[*p*]; *W* ^§^**	**[<0.000]; 0.534^§^**	**[<0.000]; 0.654^§^**	**[<0.000]; 0.380^§^**	**[<0.000]; 0.330^§^**	–
**Total protein**	**(g·dL^-1^)**	**REST**	8.73 ± 0.56^A^ (8.51 – 8.95)	8.57 ± 0.61^A^ (8.33 – 8.81)	8.77 ± 0.51 (8.58 – 8.97)	8.72 ± 0.66^A^ (8.47 – 8.98)	[0.221]; 0.055**^†^** [0.829]; 0.002**^†^** **[0.005]; 0.152^†^**
		**POST-EX**	9.05 ± 0.73^B^ (8.76 – 9.33)	8.80 ± 0.65^B^ (8.55 – 9.05)	8.99 ± 0.66 (8.73 – 9.25)	9.00 ± 0.58^B^ (8.77 – 9.22)	[0.188]; 0.059**^†^** [0.775]; 0.003**^†^** **[0.013]; 0.128^†^**
		**REC**	8.81 ± 0.70^A^ (8.54 – 9.09)	8.72 ± 0.62^B^ (8.47 – 8.96)	8.88 ± 0.76 (8.58 – 9.18)	8.80 ± 0.71^AB^ (8.53 – 9.08)	[0.637]; 0.023**^†^** [0.229]; 0.060**^†^** **[0.019]; 0.128^†^**
		**[*p*]; *η* ^2^ *_p_* ^†^**	**[<0.000]; 0.324^†^**	**[<0.000]; 0.242^†^**	[0.064]; 0.100**^†^**	**[0.012]; 0.150^†^**	–
**Albumin**	**(g·dL^-1^)**	**REST**	5.50 ± 0.36^A^ (5.36 – 5.64)	5.48 ± 0.37^A^ (5.34 – 5.63)	5.52 ± 0.37^A^ (5.38 – 5.66)	5.48 ± 0.38^A^ (5.34 – 5.63)	[0.946]; 0.005**^†^** [0.631]; 0.009**^†^** **[<0.000]; 0.229^†^**
		**POST-EX**	5.68 ± 0.45^B^ (5.51 – 5.86)	5.60 ± 0.39^B^ (5.45 – 5.75)	5.65 ± 0.43^AB^ (5.48 – 5.82)	5.63 ± 0.36^B^ (5.49 – 5.77)	[0.662]; 0.020**^†^** [0.674]; 0.007**^†^** **[0.004]; 0.158^†^**
		**REC**	5.63 ± 0.45^B^ (5.45 – 5.81)	5.62 ± 0.38^B^ (5.48 – 5.77)	5.68 ± 0.45^B^ (5.50 – 5.86)	5.64 ± 0.48^B^ (5.45 – 5.82)	[0.915]; 0.007**^†^** [0.262]; 0.052**^†^** **[0.018]; 0.129^†^**
		**[*p*]; *η* ^2^ *_p_* ^†^**	**[<0.000]; 0.243^†^**	**[<0.000]; 0.341^†^**	**[0.008]; 0.170^†^**	**[0.026]; 0.155^†^**	–
**Creatinine**	**(μmol·L^-1^)**	**REST**	105.6 ± 12.2^A^ (100.8 – 110.3)	103.9 ± 12.6^A^ (99.0 – 108.7)	104.4 ± 13.2^A^ (99.3 – 109.6)	102.1 ± 13.7^A^ (96.8 – 107.4)	[0.373]; 0.039**^†^** [0.995]; 0.000**^†^** **[0.007]; 0.144^†^**
		**POST-EX**	112.6 ± 14.7^B^ (106.8 – 118.5)	108.9 ± 13.2^B^ (103.7 – 114.0)	111.8 ± 15.7^B^ (105.8 – 117.9)	106.8 ± 13.8^B^ (101.5 – 112.2)	[0.026]; 0.116^†^ [0.777]; 0.003**^†^** **[<0.000]; 0.213^†^**
		**REC**	113.0 ± 14.7^B^ (107.2 – 118.8)	112.8 ± 14.9^C^ (107.1 – 118.6)	113.5 ± 16.5^B^ (107.0 – 120.0)	108.7 ± 14.8^B^ (103.0 – 114.5)	[0.459]; 0.035**^†^** [0.809]; 0.002**^†^** [0.083]; 0.088**^†^**
		**[*p*]; *η* ^2^ *_p_* ^†^**	**[<0.000]; 0.329^†^**	**[<0.000]; 0.396^†^**	**[<0.000]; 0.416^†^**	**[<0.000]; 0.268^†^**	–
**Urea**	**(mmol·L^-1^)**	**REST**	7.2 ± 1.9^B^ (6.5 – 8.0)	6.9 ± 1.5^B^ (6.3 – 7.5)	6.9 ± 1.4^B^ (6.4 – 7.5)	7.0 ± 1.4^B^ (6.4 – 7.5)	[0.638]; 0.021**^†^** [0.376]; 0.030**^†^** **[0.008]; 0.141^†^**
		**POST-EX**	6.9 ± 1.8^A^ (6.2 – 7.6)	6.5 ± 1.3^A^ (6.0 – 7.0)	6.5 ± 6.1^A^ (7.0 – 1.2)	6.6 ± 1.4^A^ (6.0 – 7.1)	[0.353]; 0.041**^†^** [0.423]; 0.025**^†^** **[0.015]; 0.125^†^**
		**REC**	7.2 ± 2.0^B^ (6.4 – 8.0)	7.0 ± 1.5^B^ (6.4 – 7.6)	6.9 ± 1.4^B^ (6.4 – 7.5)	6.9 ± 1.5^B^ (6.4 – 7.5)	[0.753]; 0.016**^†^** [0.435]; 0.026**^†^** **[0.035]; 0.112^†^**
		**[*p*]; *η* ^2^ *_p_* ^†^**	**[0.002]; 0.217^†^**	**[<0.000]; 0.447^†^**	**[<0.000]; 0.345^†^**	**[<0.000]; 0.288^†^**	–
**Glucose**	**(mg·dL^-1^)**	**REST**	90 ± 29^A^ (77 – 105)	92 ± 23^A^ (79 – 102)	90 ± 37^A^ (72 – 109)	89 ± 25^A^ (80 – 105)	[0.953]; 0.005**^§^**
		**POST-EX**	117 ± 29^B^ (106 – 134)	119 ± 34^B^ (101 – 135)	122 ± 20^B^ (112 – 131)	121 ± 26^B^ (105 – 131)	[0.664]; 0.024**^§^**
		**REC**	103 ± 14^B^ (98 – 112)	106 ± 25^B^ (96 – 121)	101 ± 24^A^ (90 – 114)	103 ± 15^B^ (96 – 111)	[0.094]; 0.097**^§^**
		**[*p*]; *W* ^§^**	**[<0.000]; 0.340^§^**	**[<0.000]; 0.438^§^**	**[<0.000]; 0.386^§^**	**[0.001]; 0.269^§^**	–

**^†^** The results are expressed as the mean ± standard deviation and 95% confidence interval (in parentheses). ^§^The results are expressed as the median ± interquartile range and upper – lower quartile (in parentheses). ^†^The data were analyzed with a mixed model of RM ANOVA with treatment sequence as a predictor (*treatment x sequence*) followed by the Bonferroni test; the effect size is expressed as partial eta-squared (η^2^ *_p_*). ^§^The data were analyzed with Friedman’s ANOVA followed by *post-hoc* for Friedman; the effect size is expressed as Kendall’s *W*. ^a,b^different letters refer to significant differences between study visits for simple effect of *treatment* (*COL_PRE_, COL_POST_, PLA_PRE_, PLA_POST_*). ^A,B,C^different letters refer to significant differences between measuring time points (REST, POST-EX, and REC) within the same study visit.Results in bold refer to statistically significant differences.

There were significant *treatment x sequence* interactions for: *a)* TP at REST (*p*=0.005, *η* ^2^ *_p_* =0.152; *post-hoc* did not indicate differences), POST-EX (*p*=0.013, *η* ^2^ *_p_* =0.128; *post-hoc* did not indicate differences), and REC (*p*=0.019, *η* ^2^ *_p_* =0.128; *post-hoc* did not indicate differences); *b)* ALB at REST (*p*<0.000, *η* ^2^ *_p_* =0.229; *post-hoc* did not indicate differences), POST-EX (*p*=0.004, *η* ^2^ *_p_* =0.158; *post-hoc* did not indicate differences) and REC (*p*=0.018, *η* ^2^ *_p_* =0.129; *post-hoc* did not indicate differences); *c)* CREA at REST (*p*=0.007, *η* ^2^ *_p_* =0.144; *post-hoc* did not indicate differences) and POST-EX (*p*<0.000, *η* ^2^ *_p_* =0.213) and *d)* UREA at REST (*p*=0.008, *η* ^2^ *_p_* =0.141; *post-hoc* did not indicate differences), POST-EX (*p*=0.015, *η* ^2^ *_p_* =0.125; *post-hoc* did not indicate differences) and REC (*p*=0.035, *η* ^2^ *_p_* =0.112; *post-hoc* did not indicate differences; **Table 4**).

There were exercise-induced variations in muscle damage and nutritional status markers between measuring time points (REST *vs*. POST-EX *vs*. REC) within the same visit and they are indicated in the **Table 4** by uppercase latter superscripts.

### 3.6 Carryover effect analysis

In COL→PLA sequence of supplementation subgroup there were significant differences between T_1_ and T_3_ visits in REST values of RBC (lower at T_3_ *vs.* T_1_; *p*=0.034, *d*=0.663), HTC (higher at T_3_ *vs.* T_1_; *p*=0.002, *W*=0.883), MCV (higher at T_3_ *vs.* T_1_; *p*=0.035, *d*=-0.660), RDW-S (higher at T_3_ *vs.* T_1_; *p*=0.018, *d*=-0.756), PLCR (lower at T_3_ *vs.* T_1_; *p*<0.000, *d*=1.284), TP (lower at T_3_ *vs.* T_1_; *p*=0.033, *W*=0.591), ALB (lower at T_3_ *vs.* T_1_; *p*=0.047, *d*=0.613), and CREA (lower at T_3_ *vs.* T_1_; *p*=0.019, *d*=0.751) (**Table 5**).

**Table 5:** Carryover effect analysis.

Indicator	Unit	COL→PLA *n* = 13			PLA→COL *n* = 15		
		T_1_	T_3_	[*p*]; *d* ^†^ or [*p*]; *r_c_* ^§^	T_1_	T_3_	[*p*]; *d* ^†^ or [*p*]; *r_c_* ^§^
**IgD**	**(IU·mL^-1^)**	57.8 ± 295.1 (50.1 – 345.2)	62.2 ± 110.4 (33.5 – 143.9)	[0.075]; 0.536^§^	69.2 ± 143.9 (55.5 – 199.4)	72.2 ± 67.2 (38.7 – 105.9)	**[0.020]; 0.601^§^**
**IgM**	**(g·L^-1^)**	1.09 ± 0.49 (0.86 – 1.35)	1.18 ± 0.42 (0.89 – 1.31)	[0.552]; 0.165^§^	1.15 ± 0.75 (0.79 – 1.54)	0.95 ± 0.83 (0.68 – 1.51)	**[0.011]; 0.660^§^**
**WBC**	**(10^9^·L^-1^)**	5.7 ± 1.2 (4.9 – 6.4)	5.9 ± 1.1 (5.3 – 6.6)	[0.391]; -0.247**^†^**	5.4 ± 1.2 (4.7 – 6.0)	6.3 ± 1.2 (5.6 – 7.0)	**[0.028]; -0.635^†^**
**LYM**	**(10^9^·L^-1^)**	2.2 ± 0.5 (1.9 – 2.5)	2.3 ± 0.4 (2.0 = 2.6)	[0.335]; -0.279**^†^**	2.0 ± 0.5 (1.8 – 2.3)	2.5 ± 0.4 (2.2 – 2.7)	**[0.011]; -0.756^†^**
**RBC**	**(10^12^·L^-1^)**	5.81 ± 0.27 (5.65 – 5.98)	5.73 ± 0.26 (5.57 – 5.89)	**[0.034]; 0.663^†^**	5.93 ± 0.16 (5.84 – 6.02)	5.80 ± 0.24 (5.66 – 5.93)	**[0.002]; 1.019^†^**
**HTC**	**(L·L^-1^)**	0.422 ± 0.039 (0.411 – 0.450)	0.438 ± 0.048 (0.417 – 0.465)	**[0.002]; 0.883** ^§^	0.425 ± 0.031 (0.408 – 0.443)	0.440 ± 0.031 (0.423 – 0.457)	**[0.027]; -0.638^†^**
**MCV**	**(fL)**	86.2 ± 3.9 (83.8 – 88.5)	87.4 ± 3.9 (85.0 – 89.8)	**[0.035]; 0.660^†^**	84.3 ± 2.3 (83.1 – 85.6)	86.4 ± 3.5 (84.4 – 88.4)	**[0.002]; -0.986^†^**
**MCH**	**(fmol)**	1.80 ± 0.10 (1.74 – 1.87)	1.85 ± 0.07 (1.81 – 1.89)	[0.082]; -0.526^†^	1.78 ± 0.07 (1.74 – 1.82)	1.86 ± 0.13 (1.79 – 1.93)	**[0.011]; -0.750^†^**
**RDW-C**	**(%)**	12.4 ± 1.0 (11.8 – 13.0)	12.1 ± 1.1 (11.4 – 12.7)	[0.324]; 0.285**^†^**	12.6 ± 0.9 (12.1 – 13.1)	11.8 ± 0.8 (11.4 – 12.3)	**[0.020]; 0.677^†^**
**RDW-S**	**(fL)**	39.2 ± 1.8 (38.1 – 40.3)	43.0 ± 4.1 (40.6 – 45.5)	**[0.018]; -0.756^†^**	39.1 ± 2.5 (37.7 – 40.4)	41.1 ± 3.2 (39.3 – 42.9)	[0.084]; -0.480**^†^**
**PLCR**	**(%)**	21.4 ± 7.6 (16.8 – 26.0)	13.9 ± 5.0 (10.9 – 16.9)	**[<0.000]; 1.284^†^**	24.1 ± 9.0 (19.2 – 29.1)	16.9 ± 6.0 (13.6 – 20.2)	**[<0.000]; 1.353^†^**
**Total protein**	**(g·dL^-1^)**	8.75 ± 0.55 (8.47 – 9.02)	8.41 ± 0.22 (8.34 – 8.56)	**[0.033]; 0.591** ^§^	8.86 ± 0.60 (8.62 – 9.22)	8.77 ± 0.76 (8.22 – 8.98)	**[0.004]; 0.748** ^§^
**Albumin**	**(g·dL^-1^)**	5.56 ± 0.33 (5.36 – 5.76)	5.35 ± 0.23 (5.20 – 5.49)	**[0.047]; 0.613^†^**	5.67 ± 0.40 (5.45 – 5.89)	5.44 ± 0.38 (5.23 – 5.65)	**[0.003]; 0.946^†^**
**Creatinine**	**(μmol·L^-1^)**	110.3 ± 11.8 (103.1 – 117.5)	100.9 ± 14.8 (92.0 – 109.8)	**[0.019]; 0.751^†^**	107.5 ± 11.3 (101.3 – 113.8)	101.5 ± 11.4 (95.2 – 107.8)	**[0.031]; 0.621^†^**

**^†^** The results are expressed as the mean ± standard deviation and 95% confidence interval (in parentheses). ^§^The results are expressed as the median ± interquartile range and upper – lower quartile (in parentheses). HTC, hematocrit; LYM, lymphocytes; MCH, mean corpuscular hemoglobin mass; MCV, mean corpuscular volume; MPV, mean platelet volume; PLCR, platelet large cell ratio; RBC, red blood cells; RDW-C, red blood cells distribution width – coefficient of variation; RDW-S, red blood cells distribution width – standard deviation; WBC, white blood cells. ^†^The data were analyzed with *T*-test for dependent variables; effect size expressed as Cohen’s d. ^§^The data were analyzed with the Wilcoxon signed-rank test, effect size expressed as the rank correlation coefficient (*r_c_*).Results in bold refer to statistically significant differences.

In PLA→COL sequence of supplementation subgroup there were significant differences between T_1_ and T_3_ visits in REST values of IgD (lower at T_3_ *vs.* T_1_; *p*=0.020, *W*=0.601), IgM (lower at T_3_ *vs.* T_1_; *p*=0.011, *d*=0.660), WBC (higher at T_3_ *vs.* T_1_; *p*=0.028, *d*=0.635), LYM (higher at T_3_ *vs.* T_1_; *p*=0.011, *d*=-0.756), RBC (lower at T_3_ *vs.* T_1_; *p*=0.002, *d*=1.019), HTC (higher at T_3_ *vs.* T_1_; *p*=0.027, *d*=-0.638), MCV (higher at T_3_ *vs.* T_1_; *p*=0.002, *d*=-0.986), MCH (higher at T_3_ *vs.* T_1_; *p*=0.011, *d*=-0.750), RDW-C (lower at T_3_ *vs.* T_1_; *p*=0.020, *d*=0.677), PLCR (lower at T_3_ *vs.* T_1_; *p*<0.000, *d*=1.353), TP (lower at T_3_ *vs.* T_1_; *p*=0.004, *W*=0.748), ALB (lower at T_3_ *vs.* T_1_; *p*=0.003, *d*=0.946), and CREA (lower at T_3_ *vs.* T_1_; *p*=0.031, *d*=0.621) (**Table 5**).

## 4 Discussion

To the best of our knowledge this is the first study that implemented the dose of *Colostrum Bovinum* as high as 25 g_COL_·day^-1^ for 12 weeks and comprehensively evaluated the broad range of resting, post-exercise, and recovery immunological, hematological, muscle damage and nutritional status indices in endurance-trained male athletes in a randomized crossover double-blind and placebo-controlled design. The main and the most promising result of the study is the fact, that at *COL_POST_* there was a significant POST-EX increase in salivary SIgA concentration, which remained elevated until 1 h of post-exercise recovery (REC). After COL supplementation significantly lower HTC at REC (*vs.* after PLA) was found.

As indicated above, we found that after COL supplementation, as opposed to after PLA supplementation, a significant POST-EX increase in SIgA was found, which remained significantly elevated (compared to baseline) at REC. It must be highlighted, that these alterations in SIgA concentration were exclusive to *COL_POST_* visit. SIgA plays a key role in the mucosa-associated lymphoid tissue, which forms the first line of immunological defense (29). SIgA plays a pivotal role as an antibody against respiratory pathogenic germs (29, 30). The antibodies neutralize pathogens and prevent their entry into tissues and cells, but they can also alter their surface by binding to surface proteins, which makes pathogens recognizable to phagocytic cells. SIgA is also known as an inducer of ‘active’ immunity by controlling cytokine and chemokine production (30). SIgA is also a meaningful biomarker of mucosal immunity (31). Previous studies in swimmers indicated the relationships between decreased resting SIgA concentration and increased incidence of URTI (31, 32). The latest 8-month observations in elite swimmers made by Baker et al. (31), revealed significantly lower resting absolute SIgA concentration during the weeks where upper respiratory symptoms (URS) were reported, compared to weeks free from the symptoms. Relative SIgA concentration (normalized to each individual’s mean SIgA concentration) was seen going below the individual’s ‘healthy’ level two weeks prior to URS and was about 12% lower during URS than when no symptoms present were detected (31). Although, resting SIgA concentration seems to be a relatively well-documented biomarker of URTI/URS risk, especially when analyzing its concentration in a long-term period and at the individual athlete level (31), little is known about the impact of exercise on SIgA concentration under COL supplementation. There are only four previous studies (33–36) investigating the effect of COL supplementation on post-exercise SIgA concentration in athletes, and contrary to the current investigation, none of the studies reported post-exercise improvement in SIgA concentrations as a result of COL supplementation. Still, the doses and the duration of supplementation in these investigations were lower/shorter compared to the current study – it was: 10 g_COL_·day^-1^ for 5 weeks in the study by Shing et al. (33); 20 g_COL_·day^-1^ for 4 weeks in the study by Davison and Diment (35); 10 g_COL_·day^-1^ for 8 weeks and 5 days in the study by Shing et al. (34); and 20 g_COL_·day^-1^ for 4 weeks in the study by Jones et al. (36). Thus, the results of the current study seem to be crucial, hence they indicated a unique exercise-induced response of SIgA concentration after COL supplementation. It could be hypothetically stated that COL supplementation induced a highly-specific mechanism of triggering the immune system challenged by the high-intensity exercise stimuli.

COL supplementation did not affect REST, POST-EX or REC concentrations of blood Igs (they did not increase after COL supplementation compared to pre supplementation). Apart from IgD (its POST-EX concentration at *COL_POST_* was substantially higher compared to REST and REC), there were no exercise-induced changes in blood Igs concentrations after COL supplementation. Thus, the results of the study are in line with current literature, which indicates that serum Igs remain unchanged under the influence of exercise (37). These observations are also consistent with our meta-analysis (17), where no effect of COL supplementation on pre-exercise blood concentration of IgA (based on 4 studies) or IgG (based on 5 studies) have been disclosed. Similarly, the study by Skarpańska-Stejborn et al. (38) in elite female basketball players, did not reveal the impact of 24-week COL supplementation (3.2 g_COL_·day^-1^) on resting, post-exercise, and 3-h after post-exercise recovery blood concentration of IgG compared to placebo. On the contrary, a recent study by Cieślicka et al. (39) in male football players found significantly increased resting, post-exercise and 3-h after post-exercise recovery blood concentration of IgG after 12- and 24-week COL supplementation (3.2 g_COL_·day^-1^) compared to placebo. In our study, as in line with current exercise immunology literature, in both treatments higher WBC, LYM, and MON counts were found at POST-EX than at REST and REC. Nevertheless, there were no differences between COL and PLA before/after the supplementation. Interestingly, PLA supplementation significantly increased LYM count at REST and REC. Still, there could have been a probability of disclosure of the carryover effect, since in the PLA→COL sequence subgroup, resting WBC and LYM counts were significantly higher at T_3_ (after washout and before COL supplementation) visit compared to T_1_ (before PLA supplementation) visit (WBC: 6.3 ± 1.2 *vs.* 5.4 ± 1.2 10^9^·L^-1^, *p*=0.028; LYM: 2.5 ± 0.4 *vs.* 2.0 ± 0.5 10^9^·L^-1^, *p*=0.011). In our meta-analysis (17) which included 5 studies in athletes, we did not reveal the impact of COL supplementation on pre-exercise LYM or neutrophils counts. Similarly, in the study by Skarpańska-Stejnorn et al. (38), there was no effect of 24-week COL supplementation (3.2 g_COL_·day^-1^) on resting, post-exercise and 3-h of post-exercise recovery WBC, LYM, MON and GRA counts in female basketball players.

In the current study, we found significantly lower HTC concentration at REC in *COL_POST_* than in *PLA_POST_*. Additionally, only PLA supplementation resulted in significantly reduced PLCR and RBC at REST. It must be mentioned that, there was a potential carryover effect for PLCR and RBC. The mean PLCR in both subgroups according to the supplementation sequence was significantly lower at T_3_ compared to T_1_ (T_3_ *vs.* T_1_ - COL→PLA: 13.9 ± 5.0 *vs.* 21.4 ± 7.6%, *p*<0.000; PLA→COL: 16.9 ± 6.0 *vs.* 24.1 ± 9.0%, *p*<0.000). Similarly, the mean RBC in both subgroups according to the supplementation sequence was significantly lower at T_3_ compared to T_1_ (T_3_ *vs.* T_1_ - COL→PLA: 5.73 ± 0.26 *vs.* 5.81 ± 0.27 10^12^·L^-1^, *p*=0.034; PLA→COL: 5.80 ± 0.24 *vs.* 5.93 ± 0.16 10^12^·L^-1^, *p*=0.002). Neither COL nor PLA supplementation affected the levels of the remaining studied hematological/platelet markers.

There were no differences in nutritional status and muscle damage markers after COL and PLA supplementation at any of the time points.

The undeniable strength of our approach was the utilization of crossover and long-term supplementation strategy. As far as we are concerned, only two previous studies (40, 41) on COL supplementation in athletes used a crossover design. A crossover design removes the inter-individual variability from a comparison between groups, and thus the effects of covariates are also reduced. Among the studied herein immunological markers, some of them, i.e. SIgA, are characterized by a relatively high degree of between-subject variability. To specify, so-called ‘healthy’ levels of SIgA (understood as a state free from any URS symptoms) may vary considerably between study participants (31), thus implementing a parallel group supplementation strategy, would serve as a limiting factor when analyzing and making conclusions based on means/medians at a group-level. On the other hand, a crossover design may be linked with the occurrence of the carryover effect and the impact of the sequence of treatment on the studied outcomes. In our study we utilized a randomization to the treatment sequence, so that the baseline number of participants allocated to COL→PLA and PLA→COL sequences were equal. In the final analysis, there were 13 participants allocated into COL→PLA, and 15 participants allocated into PLA→COL (with no baseline differences between sequence of treatment subgroups). Moreover, we implemented a 4-week washout period between treatment periods. In the study by Mero et al. (41) the washout period was 13 days (supplementation of 25 or 125 mL_COL/PLA_·day^-1^ for 8 days), and in the study by Carol et al. (40) it was 2.5 weeks (supplementation of 20 g_COL/PLA_·day^-1^ for 10 days). As it was mentioned before, COL contains numerous bioactive compounds. Thus, establishing a washout period is challenging, while precise information on the pharmacokinetics of particular biologically active compounds (i.e. elimination half-life) would be required. Moreover, when ingesting COL, the substances are provided in the form of a mixture, which can also affect their pharmacokinetics. On the other hand, when establishing the duration of the washout period, half-lives of biomarkers being recognized as primary outcomes were also taken into consideration. Based on the previous literature the half-life of SIgA is 3–6 days (42); IgA half-life in circulation ranges between 4 and 7 days in humans and other primates (43); IgE (44) and IgD (45) half-life in serum is 2–3 days; the serum half-life of IgG is about 21 days (43); while IgM half-life is about 5–6 days (44). Having this is mind, the implemented in our study washout period of 28 days seems to be proper for the adequate measurement of the primary outcomes of COL supplementation.

The strength of our study was the fact, that all participants started their study protocol during the autumn-winter season. Thus, the number of participants who ingested COL or PLA during autumn-winter season or spring-summer season was comparable. Under these circumstances, the number of participants at higher risk of URS (autumn-winter season) during COL and PLA supplementation was comparable. Moreover, we paid special attention to keeping exactly the same time of visits (T_1_-T_4_) of particular participants during the entire study protocol. Under these circumstances, the effect of circadian rhythm of the concentration of the studied saliva and blood markers was eliminated. It was particularly important regarding SIgA, which diurnal secretion levels are regulated by the circadian timing system and a person’s sleep-wake cycle history and actual sleepiness level (30).

One of the limitation of the current study may be the lack of the analysis of immunological outcomes after COL/PLA supplementation according to the level of energy availability of individual athletes. Recently, it has been found that fourteen days of deliberately induced low energy availability (LEA) in female athletes (22 ± 2 kcal·kg_Fat-free mass_^-1^·day^-1^) had a pronounced effect on the immune system, including increased capacity for reactive oxygen species production, altered plasma inflammatory proteome and lowered exercise-induced mobilization of leukocytes (46). In our study we did not monitor energy availability, or the relation between immunological outcomes and energy availability in study participants. Although, based on habitual diet analysis, resting nutritional status markers, or body mass/body composition evaluation we did not recognized individuals at increased risk of LEA among participants of the current study, we are convinced that the issue should be analyzed in the future studies investigating the effectiveness of COL supplementation, especially in a group of athletes particularly vulnerable to LEA.

The limitation of the current study may be connected with a supposed carryover effect for some of the studied blood markers. The carryover effect was evaluated based on the comparisons of resting concentrations of saliva and blood markers between two baseline visits – T_3_ and T_1_. Theoretically, if the implemented washout period was long enough, there should be no significant differences in the evaluated saliva and blood outcomes between T_3_ and T_1_. Nevertheless, it must be taken into account that the training loads of athletes participating in the study protocol were changing according to the individual training cycles. For instance, in our previous study in triathletes (47), we found a significant decrease in hair iron content during competition period *vs.* training period, which indicates a worsening of nutritional status during the periods of increased exercise loads. Training-induced adaptations may generate changes in saliva and blood markers. Thus, at least some of the significant differences between T_3_ and T_1_ may not be a result of the carryover effect, but a result of changes in athletes’ adaptation. From this point of view, the athletes’ population is a challenging study population. While the implemented supplementation intervention cannot interrupt the training cycles (especially), the exercise-induced effects may also be related to resting levels of saliva/blood biochemical markers. To account for seasonal changes in training loads across training cycles, we randomly assigned participants to supplementation sequences, so that the number of participants supplementing COL/PLA during training/competitive periods was nearly equal. Moreover, when verifying the inclusion criteria for participation in the study, we carefully verified training experience (at least 5 years), the number of training units per week (at least 3–5 training per week), and regular participation in triathlon/swimming competitions (at least 2–3 times per year) of volunteers. Thus, we believe that we enrolled participants characterized by relatively developed and stable physical performance and capacity (in other words we avoided enrolling athletes being at the beginning of their career and/or with short training history, to minimize the risk of rapid and dynamic changes [increase] in exercise capabilities and exercise adaptations [including ‘immuno-adaptation’] during participation in the study and their impact on the actual supplementation outcomes).

Interestingly, when analyzing differences in measured saliva and blood markers between T_3_ and T_1_ in subgroups according to the supplementation sequence, it was noted that selected nutritional status markers (total protein, albumin, RBC) and creatinine were significantly decreased at T_3_ compared to T_1_ in both subgroups (COL→PLA and PLA→COL). At the same time the concentrations of IgD and IgM were significantly decreased at T_3_ compared to T_1_ in PLA→COL, but not in COL→PLA subgroup. Thus, in both subgroups there was a clear decrease in iron- and protein-related nutritional status after cessation on the first period of supplementation, but only in PLA→COL there was a potential depletion in immunity during washout period. The latter may suggest that COL supplementation, in contrary to high-quality milk protein, might have a prolonged protective impact against immunity depletion. The observation, although interesting and promising, needs verification in the future investigations. Furthermore, one of the aspects which needs to be taken into consideration in the future investigation on COL immunological potential is energy availability, as a potential resting and exercise-induced immunomodulator.

In conclusion, after 12-week supplementation with 25 g_COL_·day^-1^ in endurance-trained male athletes a favorable increase in the post-exercise concentration of salivary SIgA was observed. Thus, COL supplementation may be useful in alleviating immune-disturbances and lowering the risk of upper respiratory tract infection arising from high training volumes and/or other stressors. Furthermore, COL supplementation had no effect on blood IgA, IgE, IgD, IgG, and IgM concentrations. Therefore, the lack of effect on blood markers indicates the need for further research in the area of mechanisms underlying the effect of COL potential immunological capacity.

## Data Availability

The original contributions presented in the study are included in the article/**Supplementary Material**. Further inquiries can be directed to the corresponding author.
