# Supplementary material for: The effect of 12-week high-dose Colostrum Bovinum supplementation on immunological, hematological and biochemical markers in endurance athletes: a randomized crossover placebo-controlled study
Source: Front Immunol. 2024 Oct 21;15:1425785. doi: 10.3389/fimmu.2024.1425785 (PMC11532120; doi:10.3389/fimmu.2024.1425785)
Supplement: Supplementary file 1 [file DataSheet1.pdf]

Supplementary Material

The effect of 12-week high-dose *Colostrum Bovinum* supplementation on immunological, hematological and biochemical markers in endurance athletes: a randomized crossover placebo-controlled study

Krzysztof Durkalec-Michalski [1,2*], Natalia Główka [1], Tomasz Podgórski [3], Małgorzata Woźniewicz [4], Paulina M. Nowaczyk [1]

[1]Department of Sports Dietetics, Poznan University of Physical Education, Poznań, Poland
[2]Sport Sciences–Biomedical Department, Charles University, Prague, Czech Republic
[3]Department of Physiology and Biochemistry, Poznan University of Physical Education, Poznań, Poland
[4]Department of Human Nutrition and Dietetics, Poznań University of Life Sciences, Poznań, Poland

*** Correspondence:**
Krzysztof Durkalec-Michalski
durkalec-michalski@awf.poznan.pl

Supplementary table 1. Nutritional value of habitual diet

Indicator	Units	COL_{PRE}	COL_{POST}	PLA_{PRE}	PLA_{POST}	[p]; W
Energy	kcal·day^{-1}	3042 ± 542 (2832 – 3252)	3102 ± 528 (2897 – 3307)	3096 ± 618 (2856 – 3336)	3074 ± 540 (2864 – 3283)	[0.702]; 0.017
	kcal·kg^{-1}·day^{-1}	37.3 ± 6.7 (34.7 – 39.9)	37.9 ± 6.7 (35.3 – 40.5)	37.9 ± 7.4 (35.1 – 40.8)	37.4 ± 6.5 (34.9 – 39.9)	[0.552]; 0.025
Protein	g·day^{-1}	139.0 ± 35.2 (125.3 – 152.6)	145.1 ± 30.2 (133.4 – 156.8)	139.1 ± 39.7 (123.7 – 154.5)	139.2 ± 30.7 (127.3 – 151.1)	[0.245]; 0.049
	g·kg^{-1}·day^{-1}	1.71 ± 0.44 (1.54 – 1.88)	1.78 ± 0.40 (1.62 – 1.93)	1.70 ± 0.45 (1.53 – 1.87)	1.69 ± 0.37 (1.55 – 1.84)	[0.228]; 0.052
Carbohydrate	g·day^{-1}	416.7 ± 84.8 (383.8 – 449.6)	429.5 ± 96.8 (392.0 – 467.0)	421.1 ± 96.0 (383.9 – 458.3)	422.5 ± 90.7 (387.4 – 457.7)	[0.062]; 0.087
	g·kg^{-1}·day^{-1}	5.11 ± 1.07 (4.70 – 5.53)	5.24 ± 1.19 (4.79 – 5.70)	5.15 ± 1.1 (4.73 – 5.58)	5.15 ± 1.08 (4.73 – 5.56)	[0.159]; 0.062
Fat	g·day^{-1}	93.0 ± 26.6 (82.7 – 103.3)	93.3 ± 29.1 (82.0 – 104.5)	93.5 ± 31.2 (81.4 – 105.6)	90.2 ± 26.4 (79.9 – 100.4)	[0.825]; 0.011
	g·kg^{-1}·day^{-1}	1.14 ± 0.32 (1.01 – 1.26)	1.14 ± 0.39 (0.99 – 1.29)	1.15 ± 0.41 (0.99 – 1.31)	1.10 ± 0.33 (0.97 – 1.23)	[0.763]; 0.014

The results are expressed as the mean ± standard deviation and 95% confidence interval (in parentheses). The data were analyzed with Friedman's ANOVA followed by *post-hoc* for Friedman; the effect size is expressed as Kendall's *W*.

Supplementary table 2. CONSORT 2010 checklist of information to include when reporting a randomised trial*

Section/Topic	Item No	Checklist item	Reported in pargraph/on page No
**Title and abstract**			
	1a	Identification as a randomised trial in the title	Yes/p. 1
	1b	Structured summary of trial design, methods, results, and conclusions (for specific guidance see CONSORT for abstracts)	Yes/p. 1
**Introduction**			
Background and objectives	2a	Scientific background and explanation of rationale	1. Introduction/p. 2-3
	2b	Specific objectives or hypotheses	1. Introduction/p. 3
**Methods**			
Trial design	3a	Description of trial design (such as parallel, factorial) including allocation ratio	2.2.Study design/ and visits/p. 4 and Figure 1
	3b	Important changes to methods after trial commencement (such as eligibility criteria), with reasons	Not applicable
Participants	4a	Eligibility criteria for participants	2.1.Study participants/p. 3
	4b	Settings and locations where the data were collected	2.1.Study participants/p. 3
Interventions	5	The interventions for each group with sufficient details to allow replication, including how and when they were actually administered	2.2.1.Supplementation/p. 4-5
Outcomes	6a	Completely defined pre-specified primary and secondary outcome measures, including how and when they were assessed	1. Introduction/p. 3<br>2.2.3.Saliva and blood collection and sample analysis /p. 5-6
	6b	Any changes to trial outcomes after the trial commenced, with reasons	Not applicable
Sample size	7a	How sample size was determined	2.1.Study participants/p. 4
	7b	When applicable, explanation of any interim analyses and stopping guidelines	Not applicable
Randomisation:			
Sequence generation	8a	Method used to generate the random allocation sequence	2.2.Study design/ and visits/p. 4
	8b	Type of randomisation; details of any restriction (such as blocking and block size)	2.2.Study design/ and visits/p. 4

Allocation concealment mechanism	9	Mechanism used to implement the random allocation sequence (such as sequentially numbered containers), describing any steps taken to conceal the sequence until interventions were assigned	2.2.Study design/ and visits/p. 4
Implementation	10	Who generated the random allocation sequence, who enrolled participants, and who assigned participants to interventions	2.2.1.Supplementation/p. 4
Blinding	11a	If done, who was blinded after assignment to interventions (for example, participants, care providers, those assessing outcomes) and how	2.2.1.Supplementation/p. 4
	11b	If relevant, description of the similarity of interventions	Not applicable
Statistical methods	12a	Statistical methods used to compare groups for primary and secondary outcomes	2.2.5.Statistical analysis/ p.6
	12b	Methods for additional analyses, such as subgroup analyses and adjusted analyses	2.2.5.Statistical analysis/ p.6
**Results**			
Participant flow (a diagram is strongly recommended)	13a	For each group, the numbers of participants who were randomly assigned, received intended treatment, and were analysed for the primary outcome	2.1.Study participants/p. 3 Figure 1
	13b	For each group, losses and exclusions after randomisation, together with reasons	2.1.Study participants/p. 3 Figure 1
Recruitment	14a	Dates defining the periods of recruitment and follow-up	2.1.Study participants/p. 3
	14b	Why the trial ended or was stopped	Not applicable
Baseline data	15	A table showing baseline demographic and clinical characteristics for each group	Table 1
Numbers analysed	16	For each group, number of participants (denominator) included in each analysis and whether the analysis was by original assigned groups	Figure 1/Table 1
Outcomes and estimation	17a	For each primary and secondary outcome, results for each group, and the estimated effect size and its precision (such as 95% confidence interval)	Figure 2, Figure 3a-e, Tables 2-4
	17b	For binary outcomes, presentation of both absolute and relative effect sizes is recommended	Not applicable
Ancillary analyses	18	Results of any other analyses performed, including subgroup analyses and adjusted analyses, distinguishing pre-specified from exploratory	Table 5
Harms	19	All important harms or unintended effects in each group (for specific guidance see CONSORT for harms)	Not applicable
**Discussion**			
Limitations	20	Trial limitations, addressing sources of potential bias, imprecision, and, if relevant, multiplicity of analyses	4.Discussion/p. 9-13
Generalisability	21	Generalisability (external validity, applicability) of the trial findings	4.Discussion/p. 9-13

Interpretation	22	Interpretation consistent with results, balancing benefits and harms, and considering other relevant evidence	4.Discussion/p. 9-13
**Other information**			
Registration	23	Registration number and name of trial registry	4. ClinicalTrials.gov (NCT06390670)
Protocol	24	Where the full trial protocol can be accessed, if available	4. ClinicalTrials.gov (NCT06390670).
Funding	25	Sources of funding and other support (such as supply of drugs), role of funders	9. Funding/p.13-14

Citation: Schulz KF, Altman DG, Moher D, for the CONSORT Group. CONSORT 2010 Statement: updated guidelines for reporting parallel group randomised trials. BMC Medicine. 2010;8:18.
© 2010 Schulz et al. This is an Open Access article distributed under the terms of the Creative Commons Attribution License (http://creativecommons.org/licenses/by/2.0), which permits unrestricted use, distribution, and reproduction in any medium, provided the original work is properly cited.

*We strongly recommend reading this statement in conjunction with the CONSORT 2010 Explanation and Elaboration for important clarifications on all the items. If relevant, we also recommend reading CONSORT extensions for cluster randomised trials, non-inferiority and equivalence trials, non-pharmacological treatments, herbal interventions, and pragmatic trials. Additional extensions are forthcoming: for those and for up-to-date references relevant to this checklist, see www.consort-statement.org.
